# *Mycobacterium smegmatis* PafBC is involved in regulation of DNA damage response

**DOI:** 10.1038/s41598-017-14410-z

**Published:** 2017-10-25

**Authors:** Begonia Fudrini Olivencia, Andreas U. Müller, Bernd Roschitzki, Sibylle Burger, Eilika Weber-Ban, Frank Imkamp

**Affiliations:** 1 University of Zurich, Institute of Medical Microbiology, Zurich, Switzerland; 2 ETH Zurich, Institute of Molecular Biology and Biophysics, Zurich, Switzerland; 3 Functional Genomic Center, University of Zurich/ETH, Zurich, Switzerland

## Abstract

Two genes, *pafB* and *pafC*, are organized in an operon with the Pup-ligase gene *pafA*, which is part of the Pup-proteasome system (PPS) present in mycobacteria and other actinobacteria. The PPS is crucial for *Mycobacterium tuberculosis* resistance towards reactive nitrogen intermediates (RNI). However, *pafB* and *pafC* apparently play only a minor role in RNI resistance. To characterize their function, we generated a *pafBC* deletion in *Mycobacterium smegmatis (Msm)*. Proteome analysis of the mutant strain revealed decreased cellular levels of various proteins involved in DNA damage repair, including recombinase A (RecA). In agreement with this finding, *Msm* Δ*pafBC* displayed increased sensitivity to DNA damaging agents. In mycobacteria two pathways regulate DNA repair genes: the LexA/RecA-dependent SOS response and a predominant pathway that controls gene expression via a LexA/RecA-independent promoter, termed P1. PafB and PafC feature winged helix-turn-helix DNA binding motifs and we demonstrate that together they form a stable heterodimer *in vitro*, implying a function as a heterodimeric transcriptional regulator. Indeed, P1-driven transcription of *recA* was decreased in *Msm* Δ*pafBC* under standard conditions and induction of *recA* expression upon DNA damage was strongly impaired. Taken together, our data indicate an important regulatory function of PafBC in the mycobacterial DNA damage response.

## Introduction

*Mycobacterium tuberculosis* (*Mtb*), the causative agent of tuberculosis, is one of the most successful human pathogens and represents a major global health problem. *Mtb* features a multitude of mechanisms that allow the phagocytozed bacteria to withstand the host’s innate and adaptive immune response and enable them to persist inside the host macrophages^1,2^. After successful immune evasion the pathogen can persist in the human for decades.

A hallmark of activated macrophages is the production of reactive oxygen species and nitric oxide, which damage bacterial proteins, lipids and nucleic acids^3,4^. Thus, in order to maintain the integrity of its genome, *Mtb* relies on robust mechanisms of DNA damage repair. Whereas in most bacteria the genes involved in these processes are regulated as part of the so-called SOS response, mycobacteria were shown to make use of an additional, independent regulatory mechanism^5–8^. In the SOS response pathway the key regulators are LexA and RecA. Under standard growth conditions the repressor LexA prevents transcription of DNA repair genes by binding to a specific sequence within their promoter, the SOS box^9^. RecA senses DNA damage by binding to single-strand DNA that is generated as a result, thereby forming nucleoprotein filaments. This in turn activates RecA to promote auto-catalytic cleavage of the repressor LexA^9^. As a consequence LexA levels initially decrease and transcription of DNA damage repair genes is de-repressed^10,11^. With only few exceptions, *Mtb* harbours the genes found in *Escherichia coli* to mediate a fully functional SOS response^12,13^.

Early studies demonstrated that the *Mtb recA* gene, which itself is regulated by LexA, contains a second, LexA-independent promoter (usually referred to as P1) located more proximal to the *recA* start codon^5–7^. Mutations in the Lex-dependent promoter P2 that abolish its activity do not abrogate basal *recA* transcription, and RecA expression remains inducible by the DNA damaging agent mitomycin C^6^. Strikingly, the majority of DNA damage inducible genes in *Mtb* are controlled in a LexA/RecA-independent manner, including also many members of the LexA-regulon^8^. In an *Mtb recA* deletion strain, induction of many DNA repair genes upon exposure to mitomycin C is either the same as in wild type or is only partly decreased. Bioinformatic analysis identified a consensus sequence in the upstream region of these genes (tTGTCRgtg - 8 nt - TannnT) that resembles the *recA* P1 promoter and is responsible for the observed LexA-independent regulation^14^. A recent study in *Msm* showed that the *clp* gene regulator (ClgR; MSMEG_2694/Rv2745c) recognizes the P1 promoter region^15^. Moreover, deletion of *clgR* in *Msm* results in impaired induction of various genes related to DNA repair mechanisms under DNA damaging conditions. This indicates a regulatory function of ClgR in the LexA/RecA-independent DNA damage repair pathway in mycobacteria.

A post-translational protein modification pathway akin to ubiquitination contributes to the survival of *Mtb* in host macrophages. In this modification pathway termed pupylation, mycobacteria (and other actinobacteria) make use of the small ubiquitin-like protein Pup to target proteins for degradation by the bacterial proteasome^16,17^. Pup is covalently attached via its C-terminal glutamate to lysine side chains of proteasomal substrates by action of the Pup ligase PafA^18,19^. Before ligation can occur, Pup, which in all mycobacteria is encoded with a C-terminal glutamine, must be rendered competent for ligation through deamidation of its C-terminal glutamine residue by the enzyme Dop (deamidase of Pup)^19,20^. Toward pupylated substrates Dop can act as a depupylase^21,22^, thereby counteracting the ligase activity. Pupylated proteins are recognized by the proteasomal ATPase ring, termed Mpa (mycobacterial proteasomal ATPase) in mycobacteria, which unfolds the substrate and translocates it into the proteolytic chamber of the proteasome complex^23–25^. The genes encoding the core proteins involved in pupylation and proteasomal degradation are clustered together in the so-called Pup-proteasome gene locus. In all mycobacterial species the gene *pafA* encoding the Pup ligase is organized in an operon together with two other genes, *pafB* and *pafC* ^26^. *Mtb* PafB and PafC were shown to copurify, suggesting that they might form a complex^26^. Deletion of the coding genes does not affect pupylation in *Mtb* and therefore has no influence on the degradation of proteasomal substrates. Unlike an *Mtb pafA* deletion mutant, a strain lacking *pafBC* displays only a slight decrease in resistance against nitrosative stress *in vitro* ^26,27^. However, PafB and PafC were linked to conjugal DNA transfer in *Msm* ^28^. Deletion of the corresponding genes does not impair the donor strain’s ability to transfer DNA. In contrast, the corresponding recipient strain is strongly compromised in DNA uptake. A recent study indicates that PafC contributes to the intrinsic resistance of mycobacteria to fluoroquinolone antibiotics^29^. However, the underlying mechanism for any of the observed phenotypes remained elusive.

In order to understand the biological function of PafB and PafC, we generated a *pafBC* deletion mutant of *Msm SMR5* and employed a comparative proteomic approach using iTRAQ (**i**sobaric **T**ags for **R**elative and **A**bsolute **Q**uantitation in mass spectrometry). Notably, a large part of the proteins that displayed decreased cellular levels in the deletion mutant are associated with DNA repair, including RecA. Accordingly, deletion of *pafBC* resulted in increased sensitivity towards DNA damaging agents, and induction of *recA* transcription in *Msm* Δ*pafBC* was strongly impaired under DNA damaging growth conditions. Strikingly, whereas the LexA/RecA-dependent *recA* promoter P2 remained inducible to wild type levels, induction of P1-driven transcription was completely blocked. Our findings establish *pafBC* as a new regulatory element in the LexA/RecA-independent DNA damage response and provide new insights into RecA regulation as well as the control of DNA repair mechanisms in mycobacteria.

## Results

### *M. smegmatis* PafB and PafC form a stable heterodimer and feature DNA binding motifs

**Figure 1:**
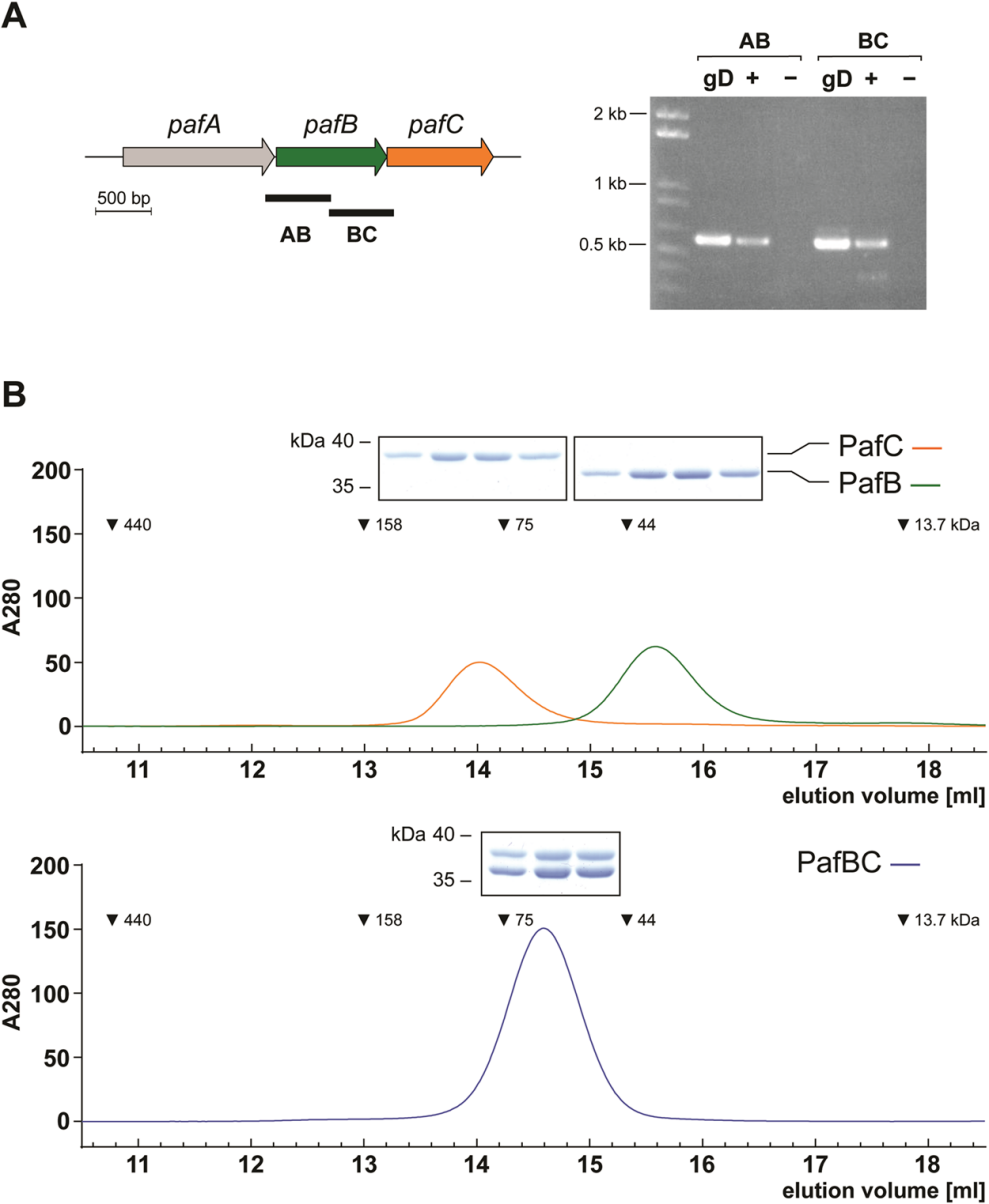
PafB and PafC form a heterodimeric PafBC complex co-expressed from a single *pafABC* mRNA. (**A**) The *pafABC* operon. Co-transcription of *pafABC* in *Msm* was confirmed by isolating mRNA from the wild type strain and a subsequent reverse transcriptase reaction. cDNA was analysed by PCR using oligonucleotides that resulted in the amplification of products spanning either *pafA* and *pafB* (AB) or *pafB* and *pafC* (BC; as indicated by black bars). PCR reactions using *Msm* genomic DNA (gD) and mRNA that was not subjected to reverse transcription (−) served as positive and negative control, respectively. (**B**) Analytical gel filtration of PafB and PafC separately (upper panel) and co-expressed and co-purified PafBC complex (lower panel). For each trace, 30 µM protein was incubated 20 min at 37 °C before running on a Superdex 200 10/300GL column at 1 ml/min at room temperature. Peak fractions analyzed by SDS-PAGE are shown as gel slices above their respective peaks.

The genes encoding PafB and PafC are located downstream of the Pup ligase gene *pafA*. Like in its pathogenic relative *Mtb* ^26^, these three genes are co-transcribed in *M*. *smegmatis* as evidenced by the presence of a single mRNA encoding both PafC and PafB in the *Msm* wild type strain (Fig. 1A). Sequence analyses of PafB and PafC showed that they exhibit similarities to a multitude of bacterial transcription factors featuring a so-called winged helix-turn-helix (wHTH) DNA-binding motif in their N-terminal region (Supplementray Fig. S1). The wHTH motif is typically comprised of three helices forming a compact helical bundle, which is capped by an adjacent hairpin (“wing”) (Supplementray Fig. S1)^30^. Binding to DNA is usually mediated by insertion of helix α3 into the major groove (Supplementray Fig. S1). Furthermore, PafB and PafC contain a C-terminal WYL domain. This domain is often found in association with wHTH motifs and is possibly involved in protein dimerization and/or the binding of ligands, which thereby trigger a specific transcriptional response^31^. Indeed, our *in vitro* analysis of the assembly state of *Msm* PafB and PafC demonstrates that the two proteins form a heterodimer (Fig. 1B). Analytical gel filtration profiles of PafB and PafC expressed and purified separately (Fig. 1B, upper panel) indicate that PafB (36 kDa) with an apparent molecular weight of 40 kDa runs as a monomer, while PafC (34 kDa) runs at an apparent molecular weight of 84 kD, possibly due to homodimer formation in absence of PafB. However, when PafB and PafC are co-expressed and co-purified (Fig. 1B, lower panel), a single main elution peak shifted away from either of the positions of the individual proteins is detected at an apparent molecular weight in agreement with a PafBC heterodimer. Consistent with this interpretation, analysis of the elution peak in SDS-PAGE reveals that it contains equal amounts of PafB and PafC. Taken together, bioinformatic analysis along with the observed stable heterodimer formation between PafB and PafC imply that the two proteins might act as a heterodimeric transcriptional regulator in mycobacteria.

### Generation of a *M. smegmatis pafBC* deletion strain

**Figure 2:**
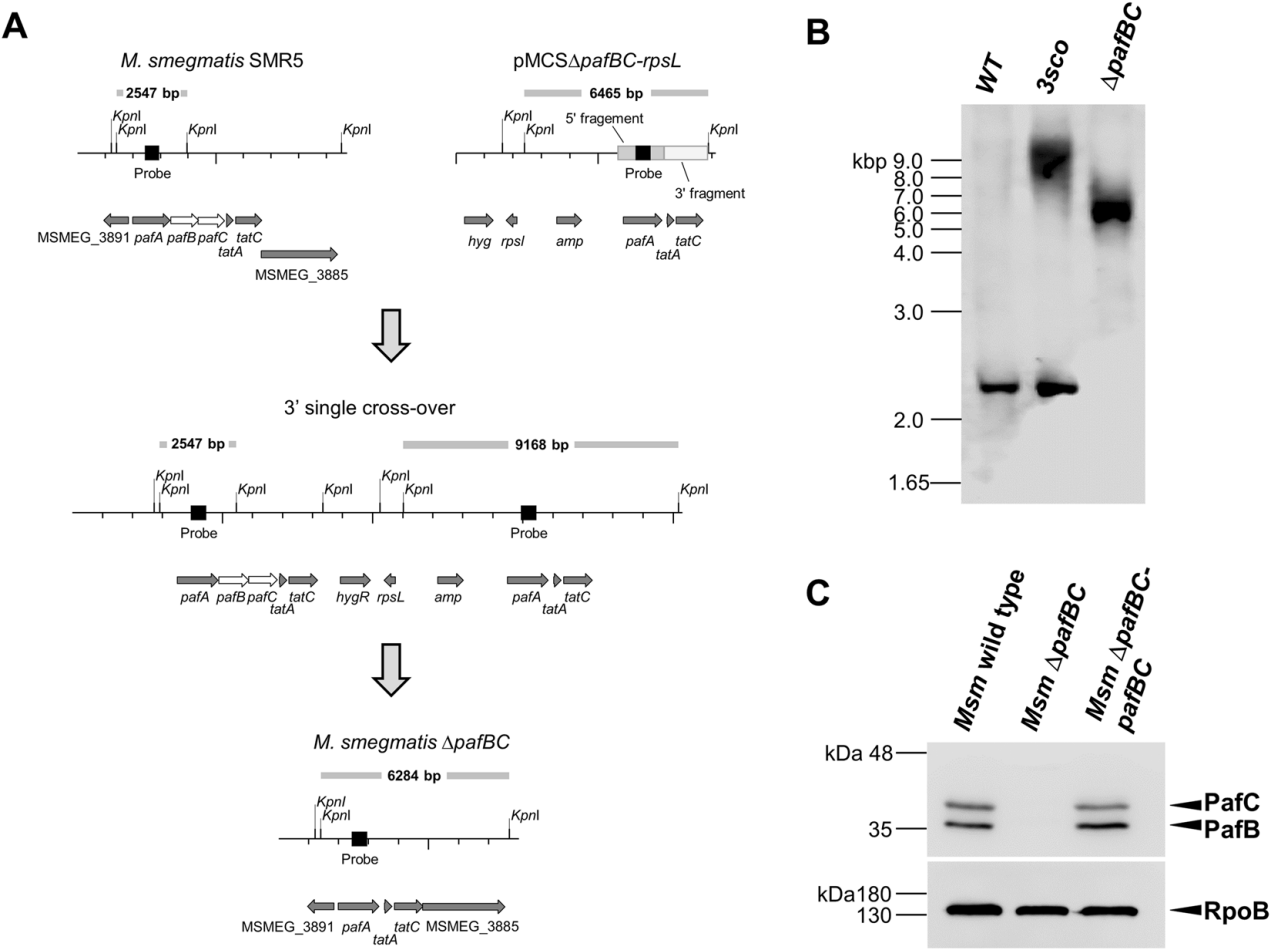
Disruption of *pafBC* in *M. smegmatis*. (**A**) Schematic representation of the generation of a markerless *pafBC* deletion mutant in *Msm*. Genes encoding PafBC are shown in white, neighbouring genes in gray. The wild type locus is shown along with the targeting vector pMCS-Δ*pafBC*, the 3’ single crossover mutant and the *Msm pafBC* deletion strain. The *pafA*-specific probe used in the Southern blot analysis is shown in black. Grey bars and sizes in bp refer to the blot shown in panel B and assign the respective fragments. (**B**) Genomic DNA of *Msm* wild type (WT), the *pafBC* 3’ single-crossover strain (3sco) and the *pafBC* deletion mutant (Δ*pafBC*) was digested with *Kpn*I. Southern blot analysis was performed using a 500-bp *pafA*-specific probe. (**C**) Anti-PafBC immunoblot analysis of *Msm* wild type, *Msm* Δ*pafBC* and the complemented strain *Msm* Δ*pafBC*-*pafBC*. The full-length blot is shown in Supplementary Fig. S6. An anti-RNA polymerase β subunit (RpoB) immunoblot served as the loading control.

As an initial step towards understanding the role of PafBC in mycobacteria we generated a markerless deletion of the *pafBC* coding genes in the model organism *Mycobacterium smegmatis* (*Msm*). The mutant was obtained by transformation of *Msm SMR5*, the streptomycin resistant parent strain carrying a point mutation in the ribosomal protein S12 coding gene (*rpsL*) with the suicide plasmid *pMCS*Δ*pafBC-hyg-rpsL* carrying the *rpsL* ^+^ gene and subsequent application of the *rpsL* counterselection strategy (Fig. 2A)^32,33^. Disruption of *pafBC* was confirmed by Southern blot analysis, and the parent strain is hereafter referred to as *Msm* wild type, the deletion strain as *Msm* Δ*pafBC*. The used probe hybridized to a 2.6-kbp-fragment in the wild type strain and to a 6.5-kbp-fragment in the *pafBC* deletion mutant (Fig. 2B). A complemented strain was generated by transforming *Msm* Δ*pafBC* with the integrative plasmid *pMV361*-*hyg*-*pafBC* carrying a fragment consisting of the presumable promoter region of the *pafABC* operon fused to *Msm pafBC*. The complementation vector re-established cellular levels of PafBC similar to wild type (Fig. 2C). *In vitro* growth of the *pafBC* deletion mutant did not differ from *Msm* wild type when grown under standard conditions at 37 °C (Supplementray Fig. S2).

**Figure 3:**
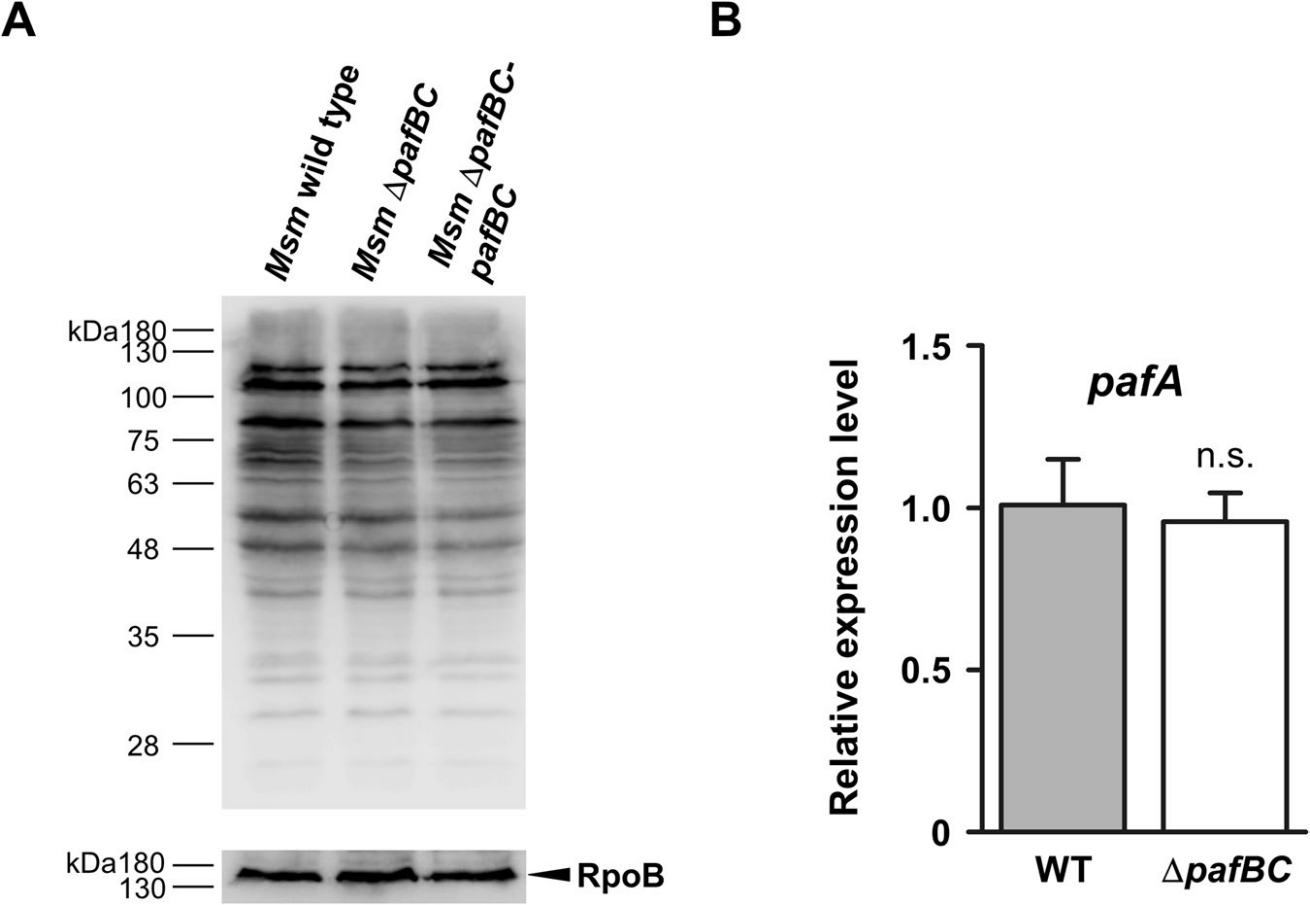
Deletion of *pafBC* does not affect pupylation of cellular proteins. (**A**) Anti-Pup immunoblot analysis of *Msm* wild type, *Msm* Δ*pafBC* and *Msm* Δ*pafBC*-*pafBC*. The full-length blot is shown in Supplementary Fig. S6. (**B**) Analysis of *pafA* transcriptional activity in *Msm* wild type and *Msm* Δ*pafBC* by quantitative real-time PCR. Values are shown as mean ± SD and represent three biological replicates measured in technical triplicates. Statistical analysis was done by an unpaired two-tailed Student’s t test; n.s., not significant.

Since *pafB* and *pafC* form an operon with the Pup-ligase encoding gene *pafA* (Fig. 1A) we investigated whether pupylation is affected by the absence of PafBC. Anti-Pup immunoblot analysis of cell-free lysates deriving from *Msm* wild type or *Msm* Δ*pafBC* grown under standard conditions revealed no significant differences in the band pattern or band intensities of the pupylated proteome (pupylome), suggesting that PafA pupylation activity remains unaffected (Fig. 3A). In accordance with this, we found that deletion of *pafB* and *pafC* did not alter the relative cellular amount of *pafA* mRNA (Fig. 3B). Similar findings were made for other proteins involved in the Pup-proteasome pathway, namely Dop, Mpa, PrcA and PrcB, which all displayed unchanged cellular protein levels in the *Msm* Δ*pafBC* mutant strain (Supplementray Fig. S3).

### Deletion of *pafBC* affects the cellular levels of proteins involved in DNA repair pathways, transcription and recombination

**Figure 4:**
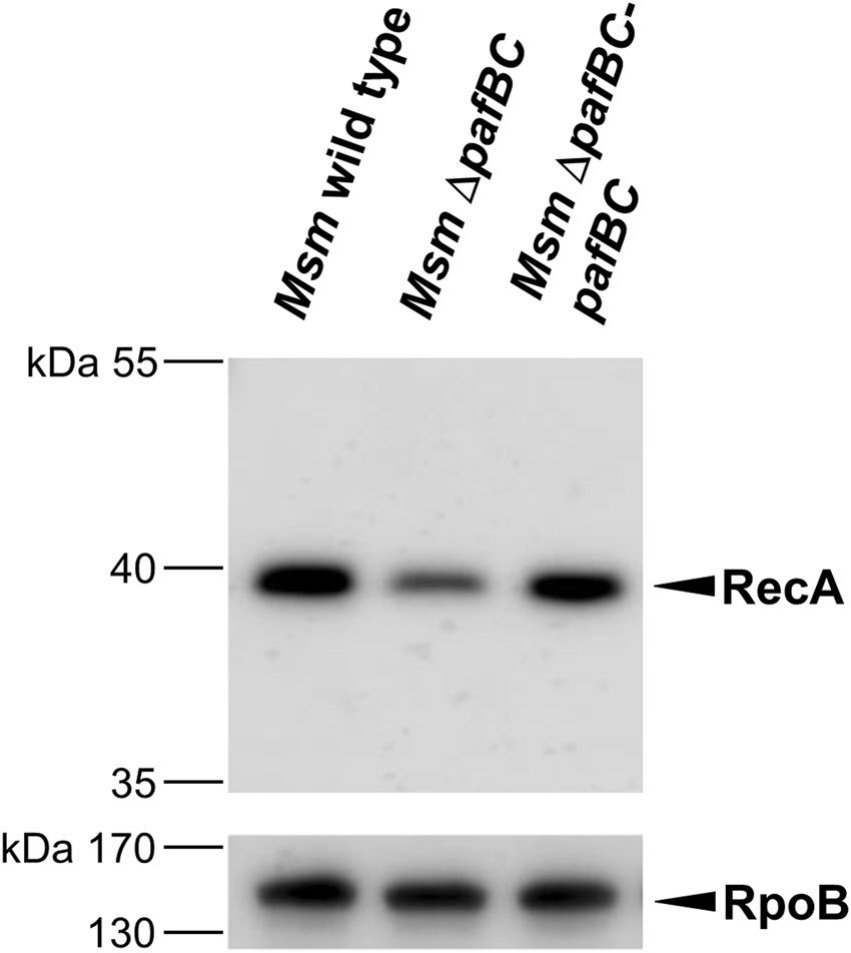
PafBC affects cellular levels of RecA in *Msm*. Analysis of RecA levels in cell-free lysates of *Msm* wild type, *Msm* Δ*pafBC* and *Msm* Δ*pafBC*-*pafBC*. Cells were grown in 7H9 at 37 °C. Cell-free lysates were analysed by immunblot using an anti-*E. coli* RecA antibody. The full-length blot is shown in Supplementary Fig. S6. Equal loading was controlled by an anti-RpoB immunoblot.

In order to gain insights into the cellular function of PafBC, a quantitative proteomic analysis of the *Msm* Δ*pafBC* mutant in comparison to the wild type strain was performed using iTRAQ. We identified a total of 2554 proteins (corresponding to 38.2% of all annotated *Msm* proteins) in three biological replicates deriving from *Msm* wild type and Δ*pafBC* cultures grown to late exponential phase. In *Msm* Δ*pafBC* 54 proteins were found to be down-regulated and 17 proteins to be up-regulated at least 1.5-fold compared to *Msm* wild type (Table 1). Among the affected proteins, 11 can be assigned to DNA-related functions, namely DNA repair, recombination and transcription. The strongest decrease was observed for RecA, with a relative reduction of about 2.5 fold in the *pafBC* deletion mutant. Accordingly, when cell-free lysates deriving from cells grown under the conditions of the iTRAQ experiment were analysed by an anti-RecA immunoblot, the signal for RecA, in the Δ*pafBC* background was significantly reduced compared to either *Msm* wild type or the complemented deletion strain (Fig. 4). Further analysis showed that RecA levels are controlled by concomitant action of PafB and PafC. Expression of either *pafB* or *pafC* alone in *Msm* Δ*pafBC* did not affect RecA expression as compared to the mutant strain, while co-expression of *pafB* and *pafC* resulted in a significant increase of cellular RecA levels (Supplementray Fig. S4).

**Table 1:** iTRAQ analysis of *Msm* wild type and *Msm* Δ*pafBC* under standard growth conditions.

Gene	Description^a^	Fold change	Gene	Description^a^	Fold change	Gene	Description^a^	Fold change
Decreased levels in *Msm* Δ*pafBC*			MSMEG_1938	Uncharacterized protein	−1.6	MSMEG_6370	AhpD	−1.5
MSMEG_0005	DNA gyrase subunit B	−1.5	MSMEG_1954	ABC1 family protein	−1.7	MSMEG_6409	Acyltransferase	−1.8
MSMEG_1252^#^	DNA helicase	−1.5	MSMEG_2198	ThiF family protein	−1.6	MSMEG_6638	Methionine synthase	−1.5
MSMEG_1327	RecB	−1.6	MSMEG_2430	Ffh	−1.7	MSMEG_6738	GntR family protein	−2.3
MSMEG_2723	RecA	−2.5	MSMEG_2667	Putative citrate lyase	−1.8	MSMEG_6759	GlpK	−1.7
MSMEG_3883	5′-3′ exonuclease	−1.6	MSMEG_2679	Uncharacterized protein	−1.8			
MSMEG_5002^##^	Conserved hydrolase	−2.0	MSMEG_3147	MoxR1	−1.6	Increased levels in *Msm* Δ*pafBC*		
MSMEG_5082	TagA	−1.8	MSMEG_3148	MoxR2	−1.7	MSMEG_6467	Dps	2.0
MSMEG_5935	RecQ	−1.9	MSMEG_3335	IclR family protein	−1.6	MSMEG_0451	Oxidoreductase	1.9
MSMEG_6153	HolB	−1.8	MSMEG_3396	IclR family protein	−1.5	MSMEG_0536	PfpI family protein	1.6
MSMEG_6939	ParA	−1.5	MSMEG_3751	Hemolysin A	−1.5	MSMEG_1356	Uncharacterized protein	2.2
MSMEG_0067	EspI	−1.5	MSMEG_3908	Uncharacterized protein	−1.6	MSMEG_1770	Uncharacterized protein	2.0
MSMEG_0064	PPE68	−1.5	MSMEG_3932	HspX	−2.3	MSMEG_1773	Uncharacterized protein	1.7
MSMEG_0224	O−methyltransferase	−1.6	MSMEG_3935	Uncharacterized protein	−1.7	MSMEG_1802	ChaB protein	2.3
MSMEG_0389	Gtf1	−1.5	MSMEG_3940	UspA	−1.5	MSMEG_1950	Uncharacterized protein	1.9
MSMEG_0408	Polyketide synthase	−1.5	MSMEG_3945	Univ. stress protein	−1.8	MSMEG_1951	Uncharacterized protein	1.9
MSMEG_0633	PAP2 family protein	−1.7	MSMEG_3959	GntR family protein	−1.5	MSMEG_2400	Ribosom. Protein L28	1.5
MSMEG_0698	IniC	−1.6	MSMEG_4121	GntR family protein	−1.6	MSMEG_2415	Uncharacterized protein	2.1
MSMEG_1244	Uncharacterized protein	−1.8	MSMEG_4524	MbtI	−2	MSMEG_5342	Uncharacterized protein	1.7
MSMEG_1352	Lipase/esterase LipG	−1.5	MSMEG_4673	ClpP1	−1.6	MSMEG_5489	Ribosom. Protein L32	1.6
MSMEG_1353	ABC1 family protein	−1.7	MSMEG_5511	von Willebrand factor	−1.6	MSMEG_5722	Uncharacterized protein	1.9
MSMEG_1366	Mkl	−1.6	MSMEG_5680	Glyoxylase family prot.	−2.1	MSMEG_6305	Uncharacterized protein	2.0
MSMEG_1671	SdhD	−1.5	MSMEG_5827	Glyoxylase family prot.	−1.8	MSMEG_6354	Serine esterase	1.7
MSMEG_1931	Uncharacterized protein	−1.6	MSMEG_5872	PhoP	−1.7	MSMEG_6755	Isoflavone reductase	1.7

Identification of regulated proteins in *Msm* Δ*pafBC* were determined using permutation test analysis and by setting filters of +/− 0.5 fold change (log_2_ scale) and a *p* value < 0.05. Fold-change values in the table are given in linear scale. **a**, descriptions are according to the UniProt database. Proteins with assigned DNA-related functions are shown in bold font. ^#^Protein is annotated as “uncharacterized” in the UniProt database. However, BLAST search^53,54^ returned “DNA helicase, **M. smegmatis**”. ^##^BLAST search excluding mycobacterial species revealed similarities to the chromosome partition protein in *Renibacterium salmoninarum* (e-value = 9e^−132^, 35% identity) and to various proteins in other actinobacterial species annotated as DNA repair ATPases.

### Down-regulation of RecA in *Msm* Δ*pafBC* occurs at the transcriptional level

**Figure 5:**
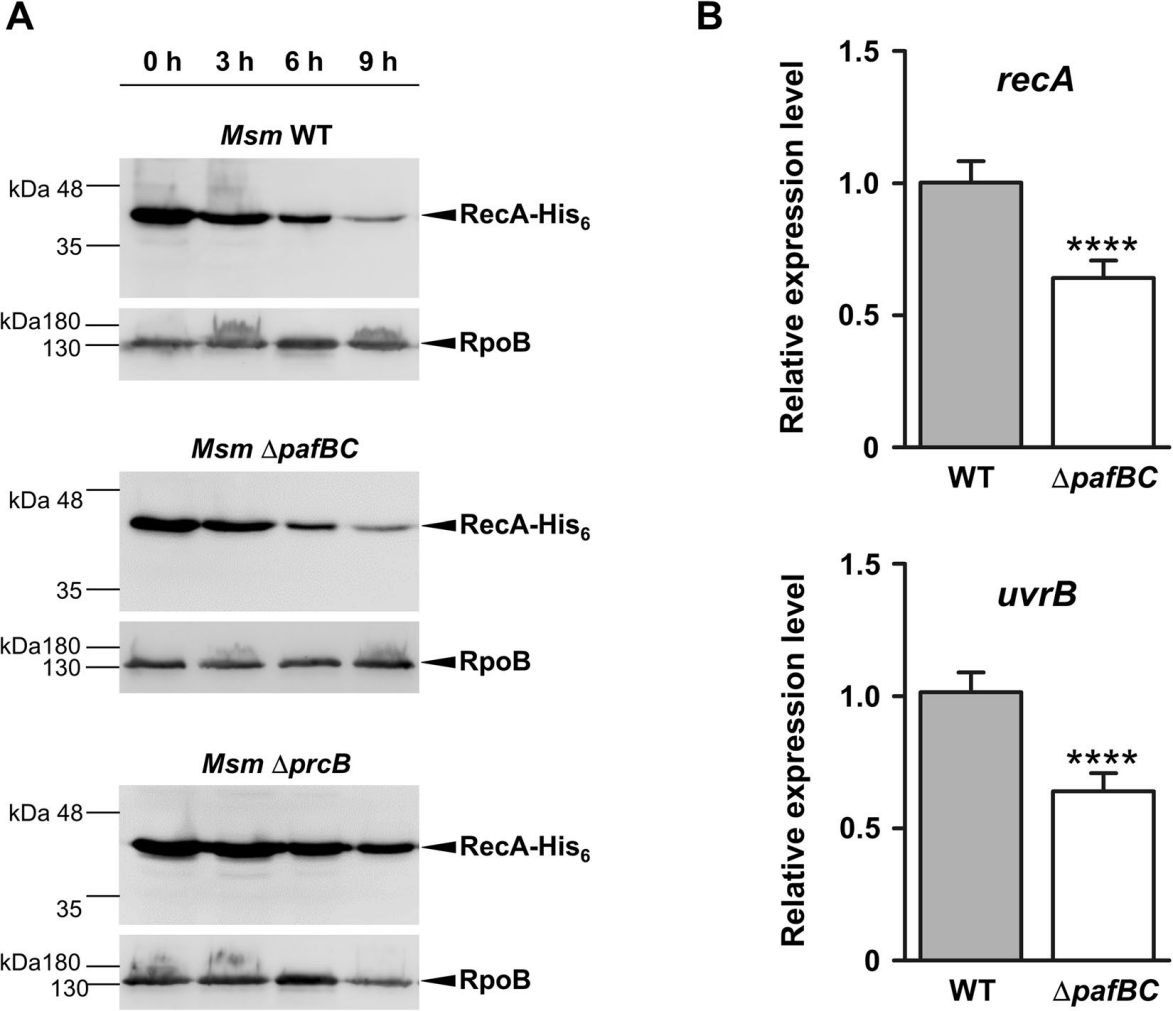
The decrease of RecA levels in *Msm* Δ*pafBC* is due to transcriptional downregulation of the coding gene and is not caused by increased proteolysis. (**A**) His_6_-RecA was expressed in *Msm* wild type, *Msm* Δ*pafBC* or *Msm* Δ*prcB* carrying a plasmid encoding *His*_6_ *-recA* gene under the control of an acetamide-inducible promoter. Cells were subsequently washed and then transferred to non-inducing growth medium. Samples were taken at the indicated time-points and turn-over of His_6_-RecA was analysed by an anti-His_6_-tag immunoblot. Full-length blots are shown in Supplementary Figure S7. Equal loading was controlled by an anti-RpoB immunoblot. (**B**) *Msm* Δ*pafBC* exhibits reduced transcription of *recA*. Values are shown as mean ± SD and represent three biological replicates measured in triplicates. An unpaired two-tailed Student’s t-test was applied for statistical analysis; ****p value < 0.001.

The changes in cellular levels of various proteins in *Msm* Δ*pafBC* implied a transcriptional regulation of the corresponding coding genes. It should be noted, however, that some of the down-regulated proteins found in our iTRAQ analysis were previously identified as possible or, like RecA, genuine targets of the pupylation pathway. In addition, we find that RecA accumulates in a proteasome-deficient strain of *Msm* (Fig. 5A, bottom blot), also indicating that RecA is degraded via the Pup-proteasome pathway in *Msm*. However, the significantly reduced levels of RecA in the *Msm* Δ*pafBC* mutant strain (Fig. 4, second lane) cannot be due to increased degradation of the protein, since *Msm* wild type and *Msm* Δ*pafBC* exhibit similar turn-over rates for a C-terminally His_6_-tagged RecA variant expressed in either of the strains (Fig. 5A, upper and middle blot). This result strongly supports the notion that transcription of *recA* in *Msm* is either directly or indirectly regulated by PafBC. Carrying out relative quantification by qRT-PCR, we found *recA* transcript levels decreased by about 36% in the *Msm* Δ*pafBC* mutant strain as compared to the parental strain (Fig. 5B, upper panel). In a concomitant control, we checked the transcriptional levels of another player involved in DNA damage repair, namely *uvrB* ^34^, whose corresponding gene product was not covered by the analysed iTRAQ samples. In analogy to *recA*, expression *uvrB* decreased by 37% in *Msm* Δ*pafBC* compared to wild type (Fig. 5B, lower panel).

Next, we examined whether PafBC influences transcription of *recA* by binding to its promoter region. To this end, an electrophoretic mobility shift assay (EMSA) was performed using purified *Msm* PafBC and a DNA fragment comprising the 300 bp upstream of the *recA* gene. Indeed, PafBC was found to bind to the *recA* promoter region (Supplementray Fig. S5). However, PafBC displays a rather promiscuous binding behaviour *in vitro* and comparable interaction with different promoter regions used as negative controls in the assay was observed (Supplementray Fig. S5). Various attempts to increase specificity by testing different buffer conditions were not successful. Therefore, the EMSA did not allow for a final conclusion concerning the binding of PafBC to the *recA* promoter region.

### Deletion of *pafBC* renders *M. smegmatis* more susceptible to DNA damaging conditions

Like in other prokaryotes, RecA in mycobacteria holds a key role in homologous recombination and was shown to be a crucial element in regulating the SOS response upon DNA damage^10,35,36^. Thus, the observed decrease of RecA prompted us to perform a phenotypical analysis of the *Msm* Δ*pafBC* mutant strain under DNA-damaging conditions.

**Figure 6:**
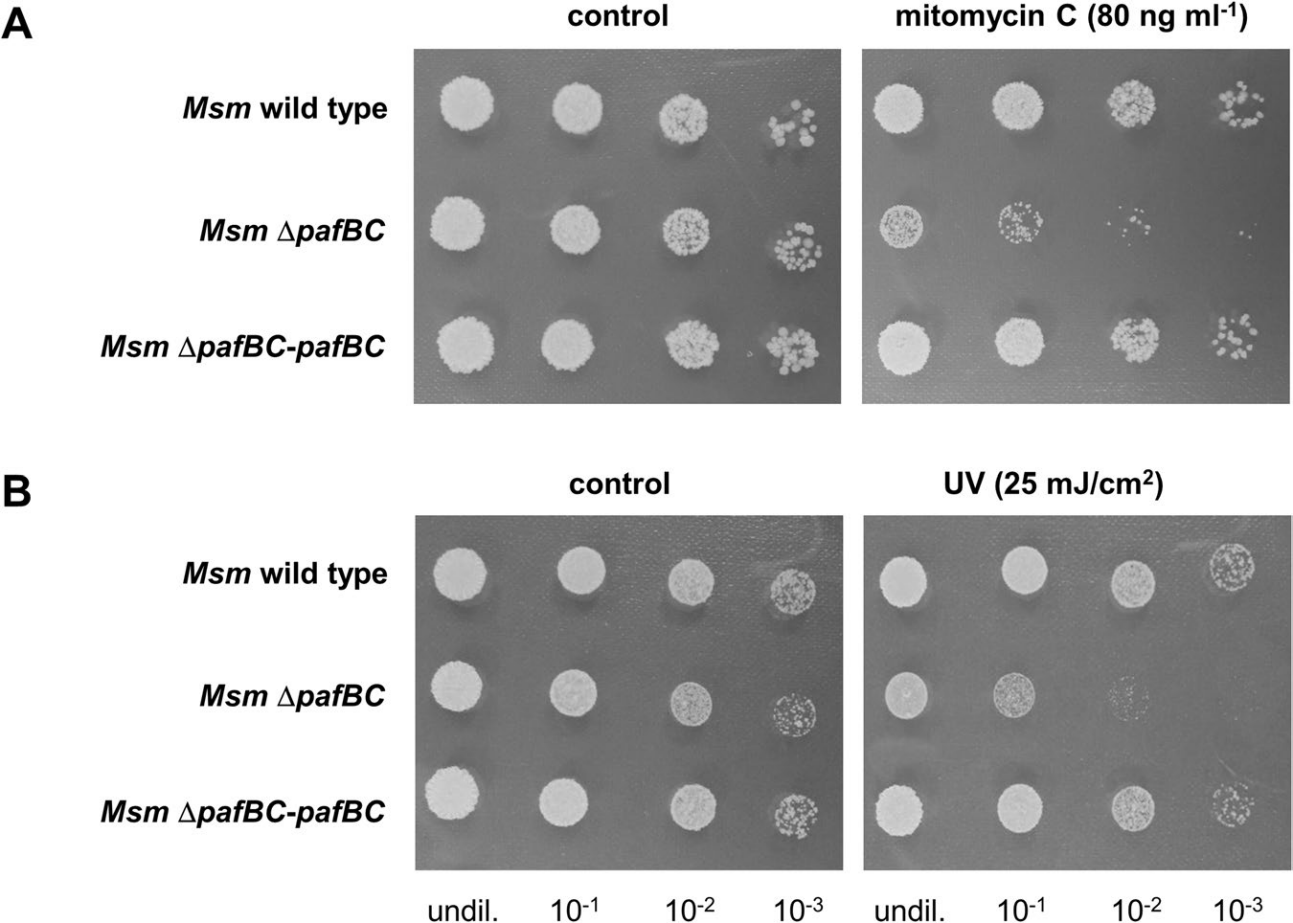
Deletion of *pafBC* in *Msm* results in increased susceptibility to DNA damaging agents. (**A**) Survival after exposure to mitomycin C. Shaking cultures were supplemented with mitomycin C at an OD_600_ of 0.1. After 8 h, serial dilutions were spotted onto LB agar plates. (**B**) *Msm* Δ*pafBC* displays increased sensitivity to UV irradiation. Survival of the strains was assessed by spotting serial dilutions of cultures grown to an OD_600_ of 1 onto LB agar plates and exposing the cells to UV light at the indicated dose.

In the growth experiments DNA stress was emulated by exposing the bacterial cultures to the DNA interstrand crosslinking agent mitomycin C. In both, *Mtb* and *Msm*, *recA* was shown to be upregulated upon exposure to mitomycin C^5^,^37,38^. In an initial approach we determined the minimal inhibitory concentration (MIC) of mitomycin C for *Msm* wild type to be 160 ng/ml and 4 ng/ml for the *pafBC* deletion strain. We subsequently performed a survival assay using 50% of the MIC for *Msm* wild type. The *Msm* wild type strain, *Msm* Δ*pafBC* and the complemented strain were grown in shaking cultures and mitomycin C was added at an OD of 0.1. After 8 h, serial dilutions of the respective cultures were spotted onto LB agar plates without mitomycin C. *Msm* wild type and the complemented strain were only mildly affected by mitomycin C, when compared to the controls grown in absence of the agent (Fig. 6A). In contrast, the number of viable cells in cultures of the *pafBC* deletion mutant decreased by about two orders of magnitude upon exposure to mitomycin C. Similar observations were made when *Msm* Δ*pafBC* was exposed to UV irradiation. After a UV dose of 25 mJ/cm^2^ viability of the deletion strain decreased by approximately two orders of magnitude as compared to the *Msm SMR5* parental strain (Fig. 6B).

### Induction of *recA* under DNA-damaging conditions is impaired in *Msm* Δ*pafBC*

**Figure 7:**
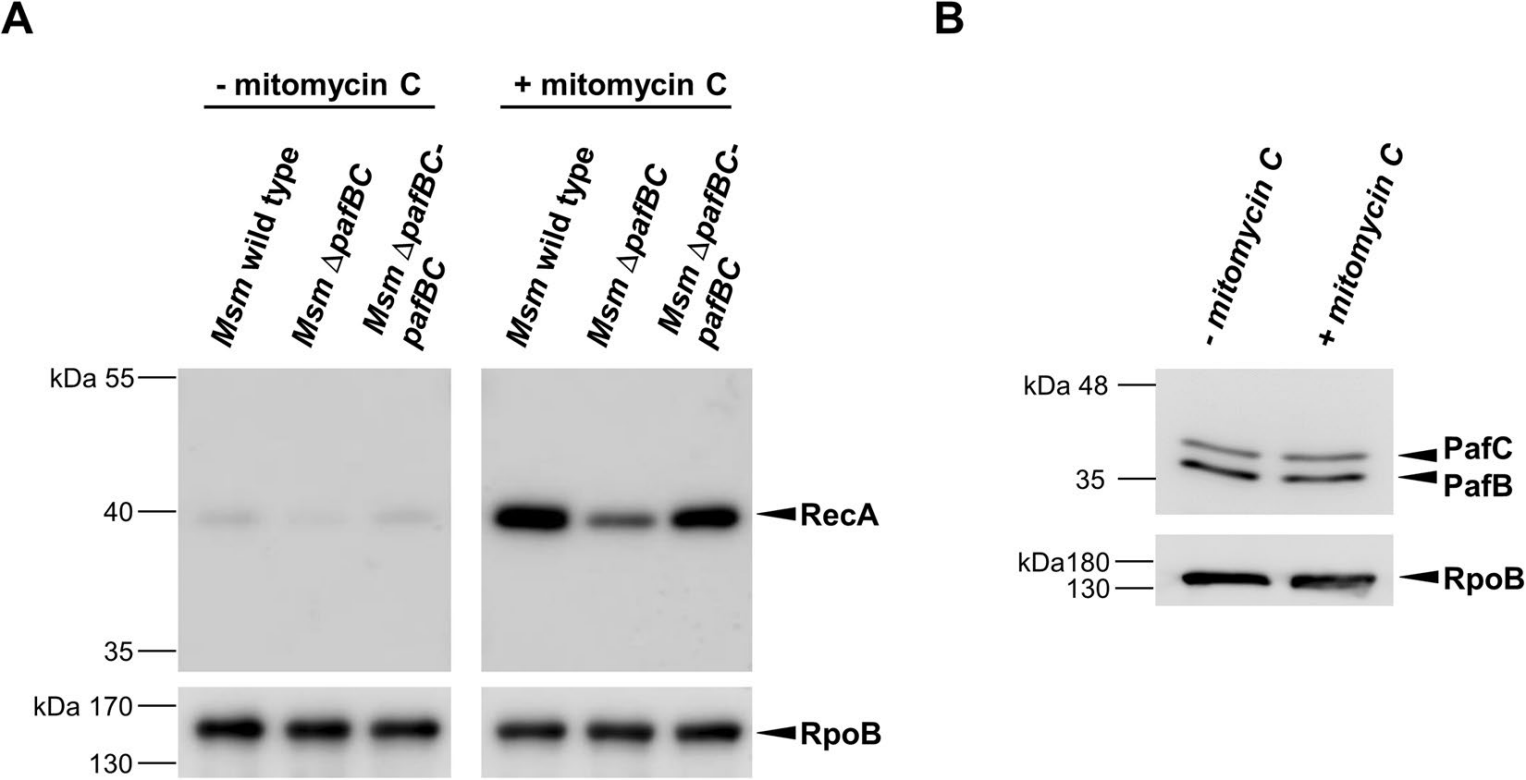
(**A**) DNA-damage induced upregulation of RecA is impaired in *Msm* Δ*pafBC*. *Msm* wild type, the *pafBC* deletion mutant and a complemented strain were grown in 7H9 to an OD_600_ between 0.8 to 1.0 at 37 °C. Cultures were split, supplemented with mitomycin C (80 ng/ml) as indicated and were incubated for another 4 h. RecA levels of cell-free lysates were analysed by an anti-RecA immunoblot. Full-length blots are shown in Supplementary Fig. S8. To control for equal loading, an anti-RpoB immunoblot was performed. (**B**) Levels of PafBC do not alter under DNA damaging growth conditions. Samples of *Msm* wild type cells were prepared as described in (**A**) and were analysed by an anti-PafBC immunoblot.

The increased sensitivity of the *Msm* Δ*pafBC* mutant towards mitomycin C and UV radiation strongly implied that the induction of *recA*, which is usually observed under DNA damaging conditions^5^, is hampered in the *Msm* Δ*pafBC* mutant. Indeed, when we performed an anti-RecA immunoblot analysis, levels of RecA increased significantly in *Msm* wild type after the exposure to mitomycin C (Fig. 7). In contrast, the *pafBC* deletion mutant exhibited significantly impaired upregulation of RecA levels under the same DNA damaging growth conditions (Fig. 7A), which could be rescued by complementation with *pafBC*. In addition, we addressed the question whether induction of *recA* under DNA damaging conditions is accompanied by upregulation of PafBC. Therefore, *Msm* wild type grown in the presence of mitomycin C was analysed by an anti-PafBC Western Blot (Fig. 7B). However, no alterations in PafBC levels were observed compared to cells grown under standard conditions.

### PafBC affects transcriptional control of *recA* via the LexA/RecA-independent promoter

**Figure 8:**
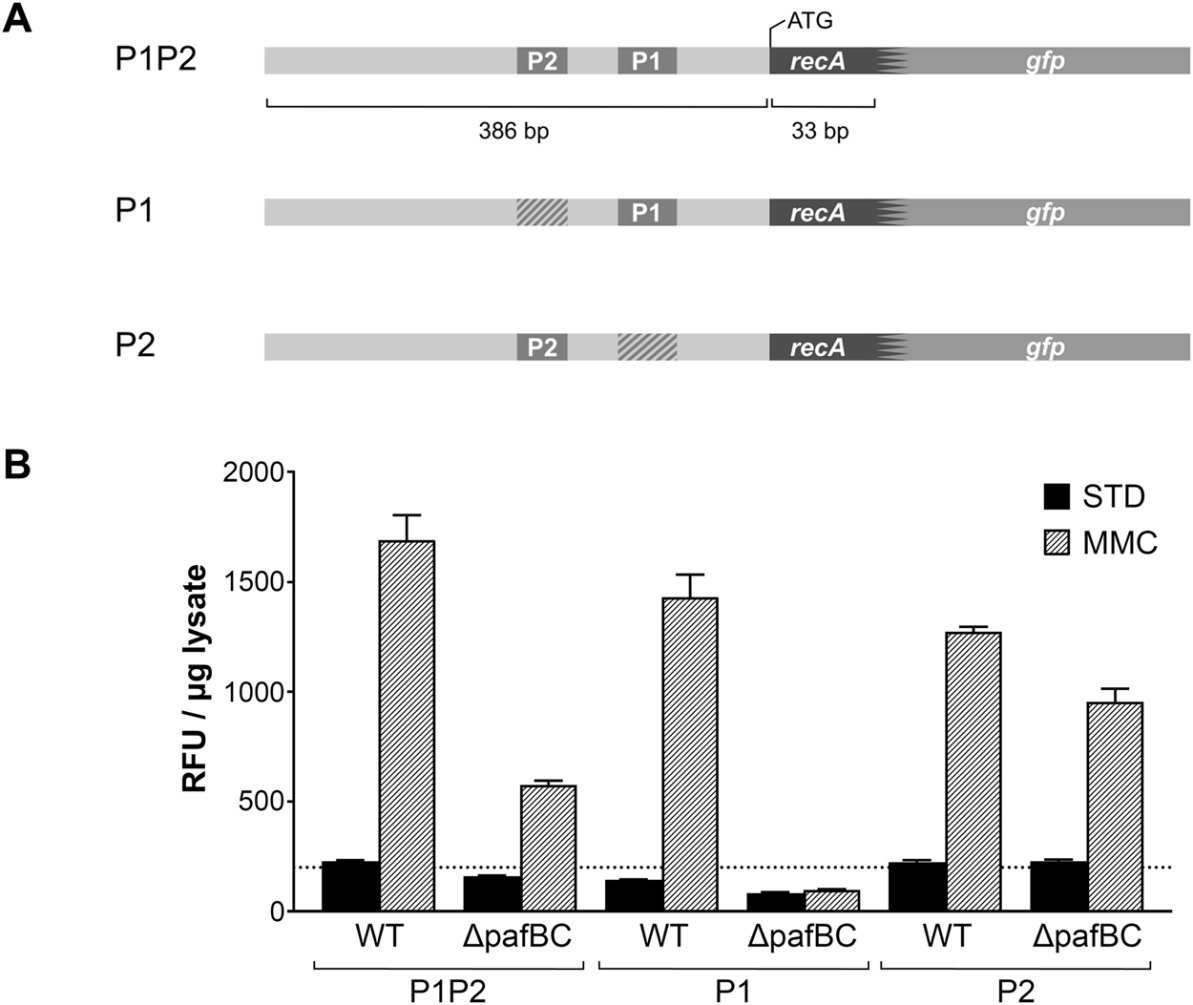
Transcriptional regulation of *recA* in *Msm* Δ*pafBC* is affected at the level of the P1 LexA/RecA-independent promoter. (**A**) Schematic representation of the *Msm recA* promoter region and the GFP reporter constructs used to measure *recA* P1, P2, and P1-P2 promoter activity, respectively. Hatched areas indicate mutated promoter regions. (**B**) *Msm* wild type and *Msm* Δ*pafBC* carrying the reporter plasmids were grown in 7H9 to an OD_600_ of 0.7 at 37 °C. Cultures were then supplemented with mitomycin C (MMC; 80 ng/ml) and were grown for an additional 4 h. Controls (STD) received no mitomycin (**C**) GFP fluorescence in lysates was measured and normalized to protein content. RFU, relative fluorescence units.

In contrast to other bacteria, *recA* is controlled by two promoters in mycobacteria: a LexA/RecA-dependent promoter (P2), which is activated as part of the classical SOS response, and a LexA/RecA-independent promoter (P1). Transcriptional activity of both, the P1 and the P2 promoter, was previously shown to be inducible by mitomycin C^6,7^. As we observed a decrease of RecA levels in *Msm* Δ*pafBC* under both standard and DNA damaging conditions, we addressed the question through which of the two promoters PafBC affects transcription of the corresponding gene. We constructed reporter strains of *Msm* wild type and *Msm* Δ*pafBC* that allowed us to monitor transcriptional activities of P1 and P2 independently. A DNA fragment encompassing the entire *recA* promoter region and the first 11 codons of *recA* was fused to GFP (Fig. 8A). Either P1 or P2 was subsequently mutated in order to determine the individual contributions of the two promoter sites with respect to the presence of PafBC. The constructs were introduced into the genome of the respective strain by an integrative vector.

In the *Msm* wild type strain reporting on the activity of the entire P1/P2 promoter region, GFP fluorescence was observed to be about 6-fold higher in presence of mitomycin C than in the absence of the agent (Fig. 8B). In the Δ*pafBC* strain the mitomycin C-induced response was markedly reduced and resulted only in an approximately 3-fold increase reaching less than half of the wild type fluorescence value. When P2 was inactivated, the response to mitomycin C stress could only be observed for the wild type strain. In contrast, mutation of P1 retained the response to mitomycin C in both strains, although the Δ*pafBC* strain displayed only about three-fourths of the wild type level. The weaker P2 response of the Δ*pafBC* strain may be explained by the fact that P2 activity is dependent on RecA^6^, whose level was shown is significantly reduced in the Δ*pafBC* strain (Figs 4A and 7A). In absence of mitomycin C, GFP fluorescence in the Δ*pafBC* strain was approximately half of the wild type level for the reporter constructs exhibiting an intact P1 promoter, while GFP fluorescence reached the same level under standard conditions when P1 was mutated. Together, these findings clearly demonstrate that P1-driven transcription of recA is absent in the pafBC deletion mutant while P2-driven transcription remains largely unaffected.

## Discussion

The response to DNA damage in mycobacteria is regulated by two independent mechanisms. Besides the ubiquitous LexA/RecA-dependent SOS response, most DNA damage-inducible genes in mycobacterial species are controlled in a LexA/RecA-independent manner via the P1 promoter^5–7^. Notably, some genes, like e.g. *recA*, are regulated by both mechanisms^8,14^. The presented study broadens the scope of P1-driven transcription of DNA repair genes by adding PafBC as a novel regulatory player to the DNA damage response in mycobacteria.

The goal of our study was to gain insights into the physiological function of *pafB* and *pafC* in mycobacteria. Since these genes form an operon with the Pup-ligase coding gene *pafA* (Fig. 1A)^26^ in mycobacteria, they were generally considered to be associated with the Pup-proteasome gene locus. The formation of a heterodimeric complex (Fig. 1B) and the presence of a winged helix-turn-helix DNA-binding motif at the N-termini of PafB and PafC (Fig. S1) indicated a regulatory function at the transcriptional level. However, under the conditions tested we found no influence of PafBC on any of the components known to be involved in pupylation or proteasomal degradation (Fig. 3, Supplementray Fig. S3). Rather, quantitative proteome analyses revealed that levels of various proteins related to DNA damage repair were altered in a PafBC-dependent manner (Table 1). Accordingly, *Msm* Δ*pafBC* displayed an increased susceptibility to DNA damaging agents (Fig. 6). Finally, using the example of RecA, we demonstrated that PafBC exerts its influence on transcription of DNA-damage inducible genes at the level of the LexA/RecA-independent P1-promoter (Figs 5B and 8). To our knowledge, this is the first study showing a role of PafBC in DNA damage response.

Disruption of *pafB* or *pafC* in *Msm* was previously shown to hamper a recipient strain’s ability to take up and/or integrate DNA via homologous recombination (HR) into its genome during conjugation^28^. RecA is a key element in HR, as it binds to the DNA strands and catalyzes strand exchange, which eventually results in recombination. In the iTRAQ analysis RecA displayed the highest decrease in cellular levels of those proteins that could be identified in the *Msm pafBC* deletion strain (Table 1). Thus, against the background of this observation the impact of disrupted *pafB* or *pafC* on conjugation can be attributed to impaired RecA-dependent HR rather than impaired DNA uptake.

Very recently, a *M. smegmatis* mutant that carries a transposon insertion in *pafC* was shown to be hypersensitive to various fluoroquinolones, like moxifloxacin or ciprofloxacin^29^. The data presented here provide an explanation for the increased sensitivity. Quinolones target topoisomerase IV and DNA gyrase and their action on these enzymes eventually results, amongst others, in multiple DNA strand breaks^39^. Thus, while *Msm* wild type is able to at least partially compensate the action of quinolones by a functional DNA damage repair, the impaired response of Δ*pafBC* to DNA damaging conditions (Figs 6 and 7) leads to increased sensitivity to these drugs.

At least under *in vitro* conditions PafBC binding lacks specificity as reflected by the results of our EMSA approach (Supplementray Fig. S4). It is possible that additional mechanisms might exist *in vivo* to bring about specificity. Many transcription factors are targeted by post-translational modifications like phosphorylation or acetylation that modulate their binding affinity^40,41^. For example, VirS, the transcription factor that regulates the mycobacterial monooxygenase (*mymA*) operon, is phosphorylated by the kinase PknK^42^. Phosphorylation increases the affinity of VirS for the *mymA* promoter. Furthermore, PafB and PafC feature a WYL domain in their C-terminal half. WYL domains were previously postulated to act in ligand sensing and binding^31^. It is therefore conceivable that PafBC relies on an additional interaction partner in order to specifically bind its genuine target, which is absent in the *in vitro* EMSA experiment. It could be argued that PafBC does not act as a specific transcriptional regulator but might function analogous to members of the Dps (DNA binding protein from starved cells) protein family^43^. Dps proteins are upregulated under various stress conditions and in several species were shown to bind DNA without apparent sequence specificity, thereby shielding the DNA molecule from damaging agents^44–46^. The fact that PafBC did not exhibit a change in cellular levels under DNA damaging conditions, makes such a mode of function very unlikely (Fig. 7B). Furthermore, most factors with wHTH motifs interact with specific DNA sequences, while DNA binding of Dps is mediated simply by the positive charge of N-terminal lysine residues^47,48^.

The involvement of PafBC in regulating the DNA damage response in *Msm* is clearly supported by our data. However, it remains elusive how the signal of DNA damage is eventually translated into PafBC-dependent transcriptional control. Regulation of DNA repair-related genes by PafBC could occur directly by binding to the P1 promoter, or indirectly by e.g. affecting the transcription of other regulators. Recently, the *clp* gene regulator ClgR was shown to recognize the LexA/RecA-independent P1 promoter^15^. Analogous to our observation with the *Msm* Δ*pafBC* mutant, upregulation of *recA* and other genes involved in DNA repair is impaired in an *Msm* Δ*clgR* mutant upon exposure to DNA damaging conditions^15^. The coding genes of 15 proteins that displayed altered levels in *Msm* upon deletion of *pafBC* either harbour the P1 consensus motif recognized by ClgR in the promoter region or are part of operons which bear the motif in their promoter region (Table S1). Based on these observations different scenarios regarding P1-driven transcription are conceivable: i) PafBC and ClgR act independently on the P1 promoter, but share certain targets, like e.g. *recA*, which are regulated by one or the other protein, depending on distinct cellular signals and/or additional transcriptional factors ii) transcription is regulated by concerted binding to the P1 promoter by both, PafBC and ClgR iii) the impact of PafBC could be indirect by affecting transcriptional activity of *clgR*.

The function of PafBC is apparently not restricted to DNA damage. This is clearly indicated by the large number of proteins (about 80% of the proteins identified in our iTRAQ analysis) whose expression is influenced by PafBC independent of the P1 promoter and that are not related to DNA repair (Table 1).This points towards a more global impact of PafBC in the cell’s regulatory networks. In accordance with an earlier study we did not find any influence of PafBC on pupylation or proteasomal degradation at least under the conditions tested (Fig. 3 and Supplementray Fig. S3)^26^. Nevertheless, the organization of *pafB* and *pafC* in an operon with *pafA* implies functional coupling and therefore a role for PafBC in the Pup-proteasome system (PPS). Interestingly, conditional expression of ClgR in *Mtb* results in the induction of *prcB* and *prcA* ^49^. Given the possible link of PafBC and ClgR indicated above, it is therefore tempting to speculate that these two regulators together might modulate levels of the PPS upon a particular cellular signal. In summary, our study assigns the first known function to the genes *pafB* and *pafC* in *Msm*. The elucidation of the function of PafBC as a heterodimeric transcriptional regulator involved in the LexA/RecA-independent mycobacterial DNA damage response adds a new facet to the intricate system of DNA damage control in mycobacteria. Yet, the co-occurrence of *pafBC* with the Pup-proteasome gene locus remains elusive. This association could have evolved since both systems - DNA repair and Pup-proteasome-dependent protein degradation - might play a role under specific stress conditions and are therefore regulated by overlapping regulatory mechanisms. Whether PafBC and the Pup-proteasome system indeed share also a more direct functional link remains to be further scrutinized.

## Methods

### Bacterial strains, oligonucleotides, media and growth conditions

The oligonucleotides used in this study are summarized in table S2. Strains used were *Mycobacterium smegmatis* (*Msm*) SMR5, *Msm* Δ*pafBC* and the complemented strain *Msm* Δ*pafBC*-*pafBC* (see following section). Bacteria were grown in LB medium supplemented with 0.05% (v/v) TWEEN-80 (LB-T) or 7H9 medium (Difco Laboratories) without OADC supplement. When appropriate, hygromycin B was added to a final concentration of 100 µg ml^−1^. Growth curves of *Msm* SMR5, *Msm* Δ*pafBC* and *Msm* Δ*pafBC*-*pafBC*, were measured in 24-well plates in a total volume of 0.5 ml. OD_600_ measurements were carried out in a Synergy H1 Hybrid Multi-Mode Microplate Reader (Biotek). For proteomic analyses, strains were grown in 7H9 medium without OADC. Cells were routinely grown at 37 °C in a shaking incubator at 140 rpm.

### Disruption of pafBC in M. smegmatis

To generate a markerless deletion of *pafBC* in *Msm* the knock-out plasmid pMCS-*hyg-rpsL*-Δ*pafBC* was generated as follows: a 1.5 kbp *Nco*I/*Hind*III fragment encompassing the 5’ *pafBC* flanking sequence and a 1.5 kbp *Hind*III/*Nde*I fragment encompassing the 3’ flanking sequence were amplified from *Msm* chromosomal DNA. The PCR products were cut with the respective restriction enzymes and were cloned into the vector pMCS-*hyg-rpsL*. Competent cells of streptomycin-resistant *Msm* mc^2^155 SMR5 were transformed with the knock-out plasmid and the *rpsL* counterselection strategy^32,33^ was applied to delete a 1.9 kbp fragment comprising *pafB* and *pafC*. Deletion of the genes was confirmed by PCR and Southern blot analysis, using a *pafA* specific 0.5 kbp probe. *Msm* Δ*pafBC* was complemented with the integrative plasmid pMV361-*hyg-amp* containing the genes encoding wild type PafB and PafC fused to 300 bp upstream sequence of the *pafABC* operon, encompassing the native promoter.

### Preparation of cell-free lysates and immunoblot analyses

Cells were harvested by centrifugation and were washed once with 1 Vol. PBS supplemented with 0.05% (v/v) TWEEN-80. Pellets were then resuspended in lysis buffer containing 100 mM Tris-HCl pH 8, 50 mM NaCl, 1 mM EDTA, 1 mM DTT, 1 mM MgCl_2_, 1 mM PMSF, supplemented with 1x protease inhibitor cocktail (Sigma-Aldrich), DNase and RNase. Cells were lysed using a FastPrep-24 Bead Beater (MP Biomedicals; 2 × 30 s at 6 m/s, 0.15mm zirconia beads). Cell debris and insoluble material was removed by centrifugation (16,000 × g, 5 min, 4 °C). Protein concentration of cell-free lysates was determined by the Bradford assay. Lysates were routinely separated by SDS-PAGE and were subjected to immunoblot analyses, using polyclonal anti-PafBC antiserum (1:1,000) raised against purified *Msm* PafBC (Eurogentec), anti-Pup antiserum (1:10,000)^19^ or anti-His_6_-tag antibody (1:10,000; Bethyl Laboratory, Montgomery, TX, USA). For detection a goat anti-rabbit IgG alkaline phosphatase antibody-horseradish peroxidase (HRP) (BioRad) or a rabbit anti-mouse IgG alkaline phosphatase antibody-HRP (Promega) was used together with Amersham ECL Prime Western Blotting Detection Reagent (1:1 ratio; GE Healthcare Life Sciences) as a substrate for the horseradish peroxidase. To control for equal loading, membranes were stripped and control immunoblots were performed using a monoclonal antibody that was raised against the RNA polymerase subunit β (RpoB) of *E. coli* (1:7,500; GeneTex). For immunoblotting of RecA, cells were harvested by centrifugation and were washed once with 700 µl PBS. Pellets were then resuspended in lysis buffer (PBS supplemented with 1 mM PMSF, 1x c0mplete (Roche) protease inhibitor), bead beaten and centrifuged as above. Lysates were separated by SDS-PAGE and were subjected to immunoblot analyses, using commercially available anti-RecA antibody (1:1,000, MBL International, clone ARM414) raised in mouse and commercially available anti-RpoB antibody (1:1,000, BioLegend, clone 8RB13) raised in mouse. For detection, HRP-conjugated anti-mouse IgG antibody (1:250,000, abcam #ab6789) together with Amersham ECL Select Western Blotting Detection Reagent (GE Healthcare Life Sciences) was used.

### Determination of the minimal inhibitory concentration (MIC)

MICs were determined as described^50^. Briefly, cultures of *Msm* SMR5 and *Msm* Δ*pafBC* were grown to mid-log phase in LB-T. Cultures were diluted to an OD_600_ of 0.01 and aliquots of 50 µl were transferred to 96-well plates containing two-fold serial dilutions of mitomycin C, resulting in a final concentration ranging from 500 to 1 ng ml^−1^. Each strain was tested in triplicates in two independent experiments. Plates were incubated at 37 °C. The MIC was defined as the mitomycin C concentration at which no visible growth was observed after three days of incubation.

### Assessing survival after mitomycin C and UV exposure

*Msm* SMR5, *Msm* Δ*pafBC* (both carrying the empty plasmid pMV361-*hyg-amp*) and the complemented strain *Msm* Δ*pafBC*-*pafBC* were grown in LB-T to OD_600_ of 1.0 and were subsequently diluted to an OD_600_ of 0.1. Cultures were supplemented with 80 ng ml^−1^ mitomycin C. Controls were grown in the absence of mitomycin C. After 8 h of incubation, serial dilutions of the cultures were spotted onto LB agar containing 100 µg ml^−1^ hygromycin B. Plates were incubated at 37 °C for 3 days and survival was assessed by comparing control samples to the samples deriving from mitomycin C-treated cultures.

For the UV irradiation assay, serial dilutions of freshly grown cultures (OD_600_ = 1.0) were spotted onto LB agar plates containing 100 µg ml^−1^ hygromycin B. Plates were exposed to a UV dose of 25 mJ/cm^2^ using a Stratalinker UV Crosslinker 2400 (Stratagene). Control samples were not exposed to UV. Plates were incubated for 3 days at 37 °C. Survival was assessed by comparing colonies grown on the respective treated and untreated plates.

### GFP-reporter assay to monitor recA P1 and P2 transcriptional activity

To monitor activities of the *recA* P1 and P2 promoter, a 419 bp DNA fragment, comprising 386 bp upstream sequence and the first 11 codons of *recA* was fused to the GFP gene and was inserted in a pMyNT-derived integrative plasmid. pMyNT was kindly provided by A. Geerlof (EMBL Hamburg). To assess the individual activity of each promoter, the P1 or P2 promoter motif was replaced with a stretch of the coding sequence of mouse histone H2Ab2 of identical size and similar GC content. The vectors were transformed into *Msm* SMR5 and *Msm* Δ*pafBC*. Three individual colonies of each reporter strain were grown in 7H9 supplemented with hygromycin B (50 µg ml^−1^) to an OD_600_ of 0.7 at 37 °C. Cultures were exposed to DNA-damaging stress by the addition of mitomycin C (80 ng/ml) and were grown for an additional 4 h. Control cultures were grown in the absence of mitomycin C. Cells were harvested by centrifugation and cell-free lysates were prepared by resuspension of the pellet in 700 µl ice-cold lysis buffer (PBS supplemented with 1 mM PMSF, 1x c0mplete EDTA-free (Roche) protease inhibitor, 1 mM EDTA) and bead beating as described above for the RecA immunoblots. Protein content was determined by the Bradford assay. GFP fluorescence was measured in a black 96-well half-area plate (30 µl lysate/well) with a Synergy2 microplate reader (BioTek) instrument (excitation filter 485/20, emission filter 528/20).

### RecA degradation assay

The gene encoding an N-terminally His_6_-tagged variant of *Msm recA* was cloned into the pMyC plasmid under the control of an acetamide-inducible promoter. *Msm* SMR5, *Msm* Δ*pafBC* and *Msm* ΔprcB carrying pMyC-His_6_-*recA* were grown in 7H9 medium to an OD_600_ of 1.0. Acetamide (final concentration 0.02% w/v) was added to the cultures to induce *recA* expression for 1 h. Cells were washed three times with PBS supplemented with 0.05% TWEEN-80 (v/v). Then cells were resuspended in 7H9 medium without acetamide and incubation of cultures continued for 9 h. Samples were withdrawn at the given time points. Cell-free lysates were prepared as described above. Equal protein concentrations were loaded and separated by SDS-PAGE for subsequent immunoblot analysis with an anti-His_6_-tag antibody in order to monitor His_6_-RecA degradation.

### RNA isolation and quantitative real-time PCR

For RNA isolation strains were grown to an OD_600_ of 2.6 in 7H9 medium without OADC. Cells were pelleted by centrifugation and were resuspended in an equal volume of Trizol-reagent (Life Technologies). The samples were subsequently lysed by bead beating using Lysing MatrixB tubes and a Fast Prep-24 device (MP Biomedicals). Supernatants were used for isolation of RNA with the RNA mini kit (Bio & Sell) according to the manufacturer’s instructions. DNA contaminations were removed by treating isolated RNA with DNase I (Thermo Fisher Scientifc). Total RNA concentration was determined by using a NanoDrop spectrophotometer (Thermo Fisher Scientific). To synthesize cDNA from *Msm* RNA the High Capacity RNA-to-DNA Kit (Applied Biosystems) was used according to the manufacturer’s instructions. Primers for qRT-PCR were designed with the Primer3 software^51^. An equivalent of ~10 ng cDNA was analysed by qRT-PCR using 5x QPCR MIX EvaGreen (Bio&Sell) in a 7500 Fast System (Applied Biosystems). Cycling conditions were as follows: 1 cycle of 95 °C for 15 min, 40 cycles of 95 °C for 15 s, 60 °C for 20 s, and 72 °C for 30 s, followed by a melting curve for each sample. cDNA derived from at least three independent biological replicates and was measured in technical triplicates, respectively. The relative expression levels were calculated according to Pfaffl^52^ using the 16 S rRNA gene as the reference.

### Proteomic analysis by iTRAQ

Cell lysates were prepared as described above from cultures of *Msm* SMR5 and *Msm* Δ*pafBC* grown to an OD_600_ of 2.5. 100 µg of proteins were digested and labelled according to the iTRAQ^TM^ manufacturing protocol (AB Sciex). A detailed description of the iTRAQ analysis can be found in the Supplementary information.

## Electronic supplementary material

Supplementary Information
