# Supplementary material for: Mycobacterium smegmatis PafBC is involved in regulation of DNA damage response
Source: Sci Rep. 2017 Oct 25;7:13987. doi: 10.1038/s41598-017-14410-z (PMC5656591; doi:10.1038/s41598-017-14410-z)
Supplement: Supplementary file 1 — Supplementary Information [file 41598_2017_14410_MOESM1_ESM.pdf]

Supplementary information

Mycobacterium smegmatis PafBC is involved in regulation of DNA damage response

Begonia Fudrini Olivencia[1#], Andreas U. Müller[2#], Bernd Roschitzki[3], Sibylle Burger[1], Eilika Weber-Ban[2], Frank Imkamp[1]*

[1]University of Zurich, Institute of Medical Microbiology, Zurich, Switzerland
[2]ETH Zurich, Institute of Molecular Biology and Biophysics, Zurich, Switzerland
[3]Functional Genomics Center Zurich, UZH/ETH Zurich, Switzerland

*Corresponding author: imkamp@imm.uzh.ch
[#]Authors contributed equally

Methods

Modelling PafB and PafC helix-turn-helix motif

Structural models of the *Msm* PafB and PafC helix-turn-helix motifs were generated using the SWISS-Model online server[1], applying the Alignment Mode. The transcriptional regulator TM1602 of *Thermotoga maritima*[2] was selected as the modelling template.

Purification of PafB, PafC, and co-expressed PafB and PafC

Genes encoding PafB (MSMEG_3889), PafC (MSMEG_3888) and a fragment encompassing the genomic arrangement PafB-PafC were amplified by PCR from *Msm* SMR5 genomic DNA using the corresponding primers listed in table S2 and were cloned into a modified pET28 vector. Constructs were expressed in *E. coli* Rosetta with an N-terminal His_6-TEV tag (in case of the PafBC co-expression construct, only PafB was tagged) at 18 °C overnight after induction with 0.5 mM IPTG. Cells were harvested and pellets were used either directly or were once resuspended in PBS, harvested again and stored at -20 °C until further processed. Pellets were resuspended in 50 mM HEPES-NaOH, pH 7.8/4 °C, 300 mM NaCl. Cells were subsequently lysed using a Microfluidizer M-110L instrument (11,000 psi chamber pressure, 5 passages). Insoluble material was removed by centrifugation (48,000 x g, 30min, 4 °C). DNase I (50 U/ml final) and imidazole (10 mM final) were added to the cleared lysate and incubated for 30min on ice. Proteins were enriched by passing the lysate through a 0.45 µm filter onto a nickel-charged resin (IMAC Sepharose 6 Fast Flow, GE Healthcare Life Sciences) and eluting with increasing imidazole concentrations. Protein-containing fractions were pooled, histidine-tagged TEV protease was added to a molar ratio of 1:30 and the solution was dialyzed overnight in 25 mM HEPES-NaOH, pH 7.8/4 °C, 50 mM NaCl, 1 mM DTT, 1 mM EDTA at 4 °C in a 12-14 kDa cut-off membrane (SpectraPor). $MgCl_2$ was added to a final concentration of 2 mM and TEV protease was removed by passing the solutions over a nickel-charged resin. PafB and PafC proteins were desalted into 50 mM HEPES-NaOH, pH 7.8/RT, 150 mM KCl, 0.5 mM TCEP, 5% v/v glycerol. Co-expressed PafB and PafC were further purified by ion exchange chromatography on a Source 30Q column (GE Healthcare Life Sciences; low salt buffer: 25 mM HEPES-

NaOH pH 7.8/4 °C, 50 mM NaCl; high salt buffer: 25 mM HEPES-NaOH pH 7.8/4°C, 1M NaCl) followed by gel filtration on a Superose 12 prep grade column (GE Healthcare Life Sciences; buffer: 10 mM HEPES-NaOH pH 7.9/4 °C, 50 mM NaCl, 0.5 mM TCEP). Proteins were concentrated using Amicon Ultra 10K centrifugal filtration devices (Merck Millipore; 3500 x g, 10 min intervals, 4 °C). Protein concentrations were determined spectrophotometrically using the calculated extinction coefficients for the reduced proteins according to the Edelhoch method[3] with the values for Trp and Tyr determined by Pace *et al*.[4]

Assaying complex formation of PafB and PafC by gel filtration

Reactions containing 30 µM protein in 50 mM HEPES-KOH, pH7.8/RT, 500 mM NaCl, 5 mM DTT were incubated at 37 °C for 20 min prior to gel filtration on a Superdex 200 10/300GL column (GE Healthcare Life Sciences) run at 1 ml/min at room temperature in 50 mM HEPES-KOH, pH 7.8/RT, 500 mM NaCl. Peak fractions were collected according to the A_{280} signal and analyzed by SDS-PAGE. Molecular weight standards (Ferritin 440 kDa, Aldolase 158 kDa, Conalbumin 75 kDa, Ovalbumin 44 kDa, RNase A 13.7 kDa) were run separately to calibrate the column.

Electrophoretic mobility shift assay

DNA fragments comprising the region 300 bp upstream of the start codon of *M. smegmatis recA* (*MSM*EG_2723), *pafA* (*MSM*EG_3890), *sucB* (*MSM*EG_4283) and *groEL2* (*MSM*EG_1583) were amplified by PCR, respectively. Binding reactions were performed by incubating increasing amounts of purified PafBC-Strep (0, 2.75, 5.5, 11 µM) with constant amounts of DNA (0.125 µM) in binding buffer (50 mM HEPES-KOH pH 7.8, 175 mM NaCl, 50 mM KCl, 1 mM $MgSO_4$, 1 mM K_2HPO_4, 10 mM imidazole, 1 µM $ZnSO_4$, 0.25 mM DTT) for 1.5 h at 25 °C. The samples were mixed with 5x loading buffer (40% sucrose, 0.25% bromophenol blue) and analysed by native PAGE. DNA and DNA/protein complexes were visualized by staining the gels with ethidium bromide.

Proteomic analysis by iTRAQ

Cell-free lysates of *Msm* SMR5 and *Msm* Δ*pafBC* grown to an OD_{600} of 2.5 were prepared. Subsequently, 100 µg of protein were denatured with 0.1% SDS and reduced with 5 mM TCEP. 10 mM methyl methanethiosulfonate (MMTS) were used as a cysteine blocking reagent. Protein samples were digested with trypsin (Promega) for 2.5 h at 37 °C and subsequently labelled with 4plex-iTRAQ^{TM} Reagent for 1.5 h at room temperature. Phosphoric acid was added to stop the reaction. Samples were combined and dissolved in 100 µl 8 mM KH_2PO_4, 75% acetonitrile (ACN), pH 4.5 by hydrophilic interaction liquid chromatography (HILIC)YMC-Pack Polyamine II column (250 x 3 mm, 5 µm, YMC Europe GmbH) connected to an analytical HPLC (LC1100, Agilent Technologies). Peptides were eluted with an increasing gradient of solvent B (100 mM KH_2PO_4, 5% ACN, pH 4.5; 0 - 50% solvent B in 30 min; 50-100% solvent B in 5 min) with a flow rate of 0.5 ml/min and collected in fractions. Pooled fractions were vacuum concentrated, dissolved in 3% ACN, 0.1% trifluoroacetic acid and desalted using ZipTips C18 (Millipore). After a further vacuum concentration step, peptides were dissolved in 3% ACN, 0.1% formic acid. Dissolved samples were injected into an Eksigent-nano-HPLC system (Eksigent Technologies) by an autosampler and separated on a self-made reverse-phase column (75 µm x 160 mm) packed with C18 material (3 µm, 200 Å, AQ, Bischoff GmbH). The column was equilibrated with 98% solvent A (A: 0.1% formic acid in water) and 2% solvent B (B: 0.1% formic acid in ACN). Peptides were eluted using the following gradient: 0-120 min; 2-38% B, 120-120 min; 38-98% B at a flow rate of 0.3 µL/min. High accuracy mass spectra were acquired at an AB Sciex 5600 (AB Sciex) in the mass range of 385-1250 m/z. Up to 36 data dependent MS/MS were recorded in high sensitivity mode (mass range 100 m/z to 1800 m/z) of the most intense ions with charge state 2+ to 5+ using collision induced dissociation. Target ions already selected for MS/MS were dynamically excluded for 60 s after three accuracies. After data collection, the peak lists were generated and analysed using Mascot 2.4 (Matrix Science, Boston, MA, USA) and searched against a decoyed *Mycobacterium smegmatis* database from UniProt (generated in January 2013) concatenated to 260 known mass spectrometry contaminants. Parameters: precursor ion mass tolerance 20 ppm, fragment ion mass tolerance of 0.1 Da, trypsin digestion, fixed modifications of MMT-labeled cysteine, 4-plex iTRAQ modifications of free amines at the N-termini and of lysine; and variable modification 4-plex iTRAQ of tyrosine and acetyl

modification at the N-termini of proteins. Scaffold_4.1 (Proteome Software Inc., Portland, OR) was used to validate MS/MS-based peptide and protein identifications. We identified 2554 proteins (protein prophet probability 95%, one peptide for identification of protein and minimum Mascot ion score of 40). Differentially expressed proteins were determined by setting a filter of +/- 0.5 fold change (log_2 scale) and a p value < 0.05.

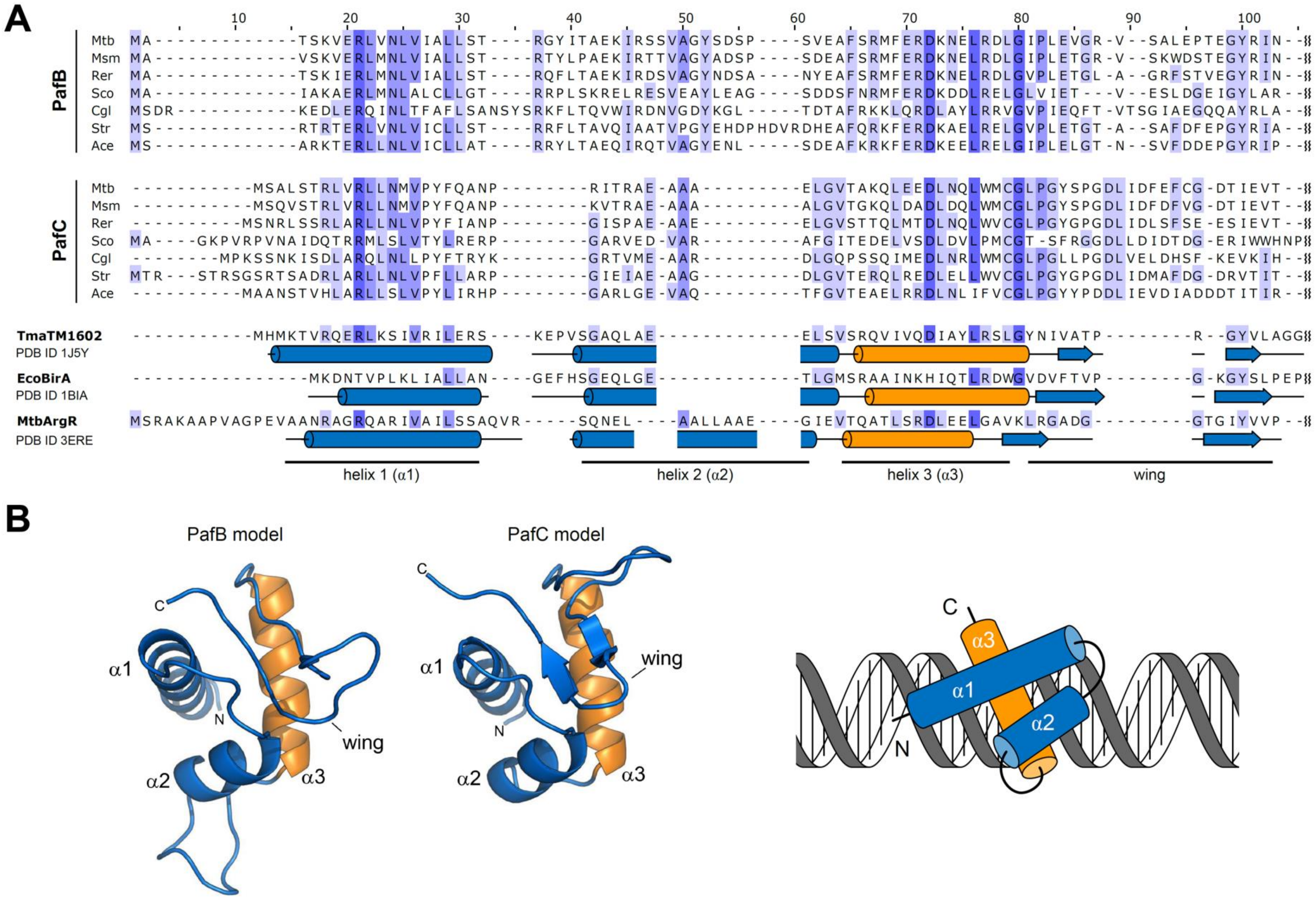

Figure S1. PafB and PafC feature an N-ternimal winged helix-turn-helix domain (wHTH). A. Protein sequences of PafB and PafC from various actinobacteria are aligned to sequences of proteins known to feature a wHTH domain: TM1602 (PDB ID 1J5Y)[2], BirA (PDB ID 1BIA)[3] and ArgR (PDB ID 3ERE)[4] (full length sequences were aligned using ClustalOmega[5]; only the first 105 alignment positions are shown). Residues are shaded according to their percent identity. Secondary structure information from the crystal structure models of the wHTH proteins is shown below the sequence. B. Homology models for wHTH motifs of PafB and PafC and schematic representation of the binding of a HTH motif to the major groove of DNA. Helix α3, primarily mediating DNA-binding and recognition of HTH domains[6] is shown in orange. Mtb, *Mycobacterium tuberculosis*, Msm, *Mycobacterium smegmatis*, Rer, *Rhodococcus erythropolis*, Sco, *Streptomyces coelicolor*, Cgl, *Corynebacterium glutamicum*, Str, *Salinispora tropica*, Ace, *Acidothermus cellulolyticus*, Tma, *Thermotoga maritima*, Eco, *Escherichia coli*

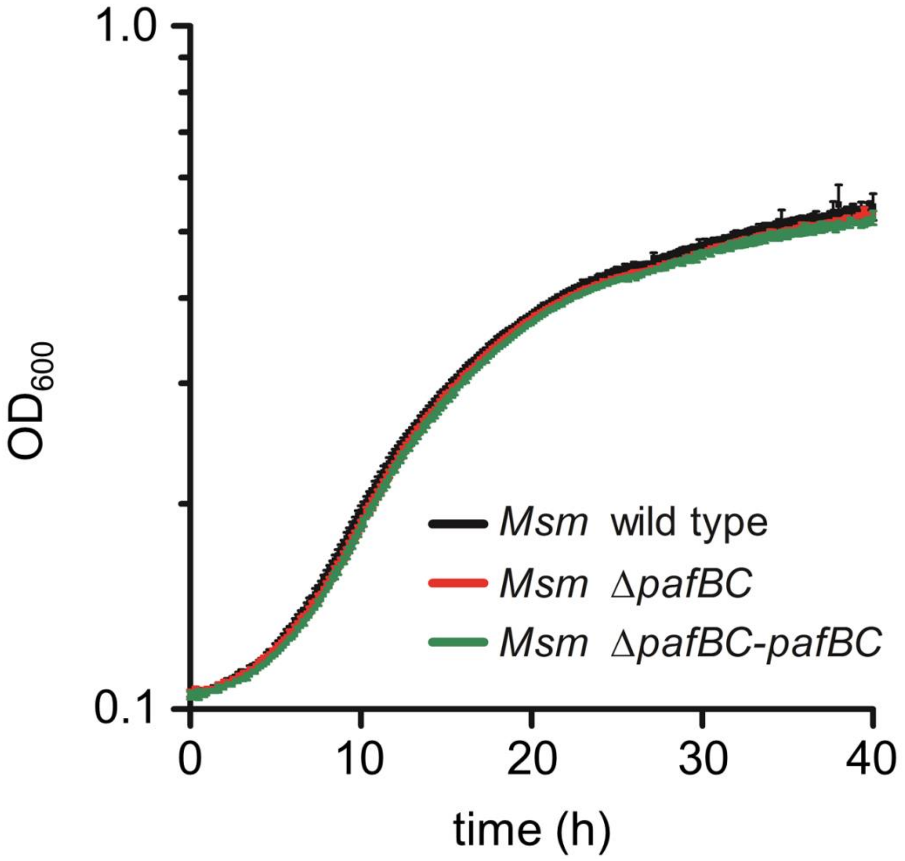

Figure S2. Growth curve for *Mycobacterium smegmatis* (*Msm)* wild type, *Msm* ∆*pafBC* and the complemented strain *Msm* ∆*pafBC-pafBC* in 7H9 medium at 37°C. OD values are shown as mean ±SD and represent three independently grown cultures, respectively.

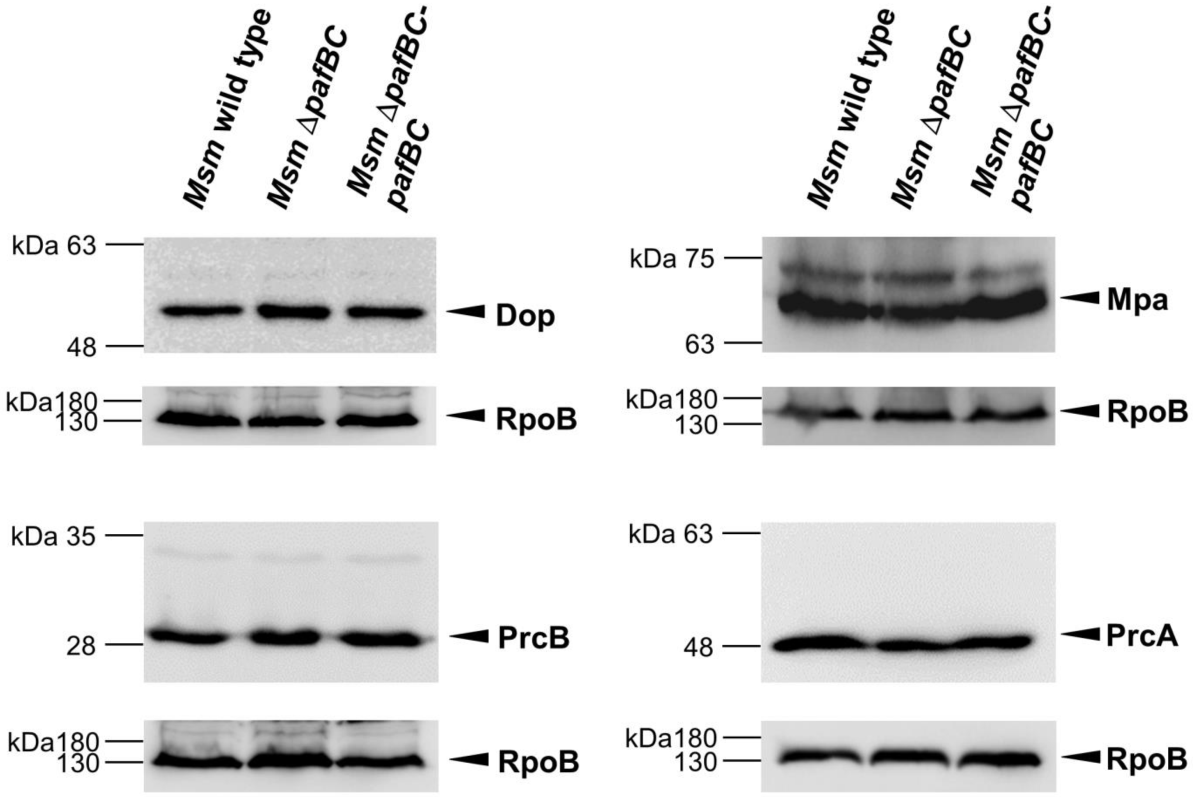

Figure S3. Immunoblot analyses of cellular levels of components involved in the Pup-proteasome system in *Msm* wild type, *Msm* Δ*pafBC* and *Msm* Δ*pafBC*-*pafBC*. Cells were grown in 7H9 medium. Cell-free lysates were analysed with antisera specific for Dop, the proteasomal chaperone Mpa and the proteasome's α and β subunits. RpoB served as the loading control.

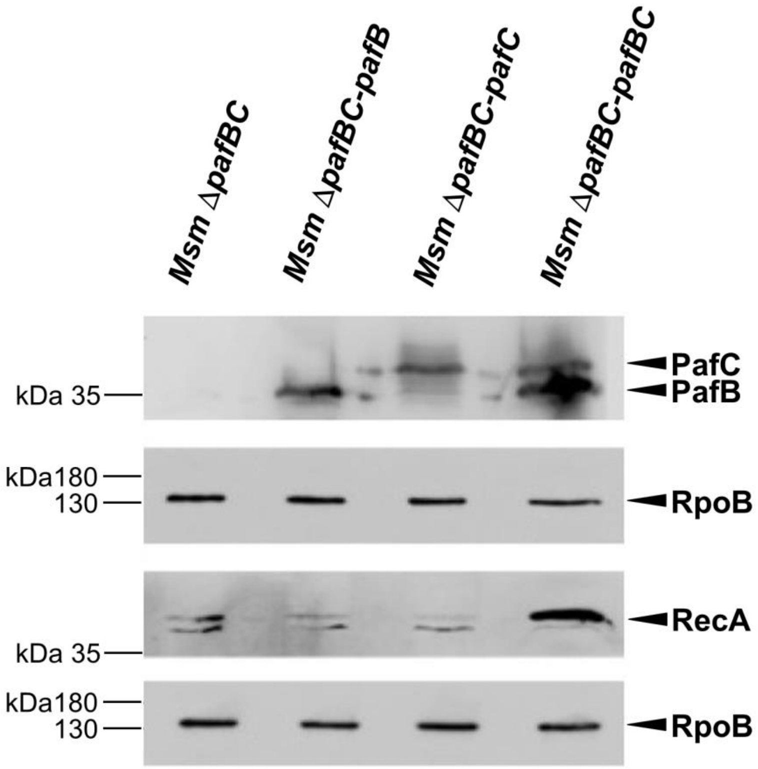

Figure S4. RecA levels in *Msm* are regulated by concomitant action of PafB and PafC. Cultures of *Msm ΔpafBC* carrying a plasmid encoding either *pafB*, *pafC* or *pafBC* under the control of an acetamide-inducible promoter were grown in LB-T in the presence of 0.4% acetamide to induce gene expression. Cells were harvested and cell-free lysates were prepared. Lysates were analysed by immunoblot using PafBC-specific and RecA-specific antibodies. RpoB served as the loading control.

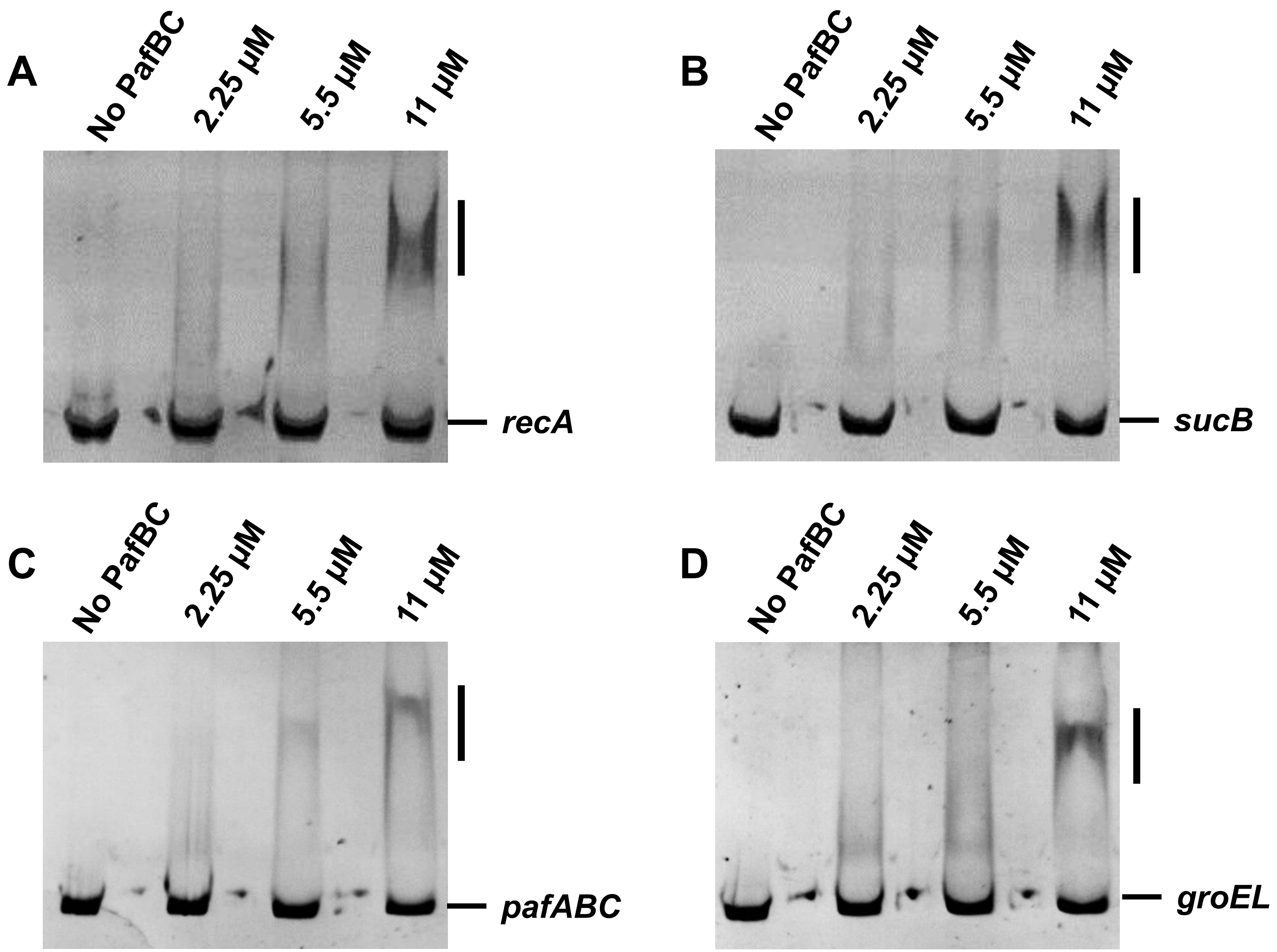

Figure S5. PafBC binds unspecifically to DNA under *in vitro* conditions. (A) The binding of *Msm* PafBC to the *recA* promoter region was investigated by EMSA. A 300-bp-DNA fragment of *recA* upstream sequence encompassing the promoter region (final concentration 0.125 µM) was incubated with increasing amounts of *Msm* PafBC (as indicated). As a control the assay was performed with the promoter regions of (B) *sucB* (MSMEG_4283), (C) the *pafABC* operon (MSMEG_3890/89/88) and (D) *groEL* (MSMEG_1583). Unbound DNA and DNA/protein complexes were separated by PAGE and were subsequently stained with ethidiumbromide. Black bars indicate DNA/PafBC complexes.

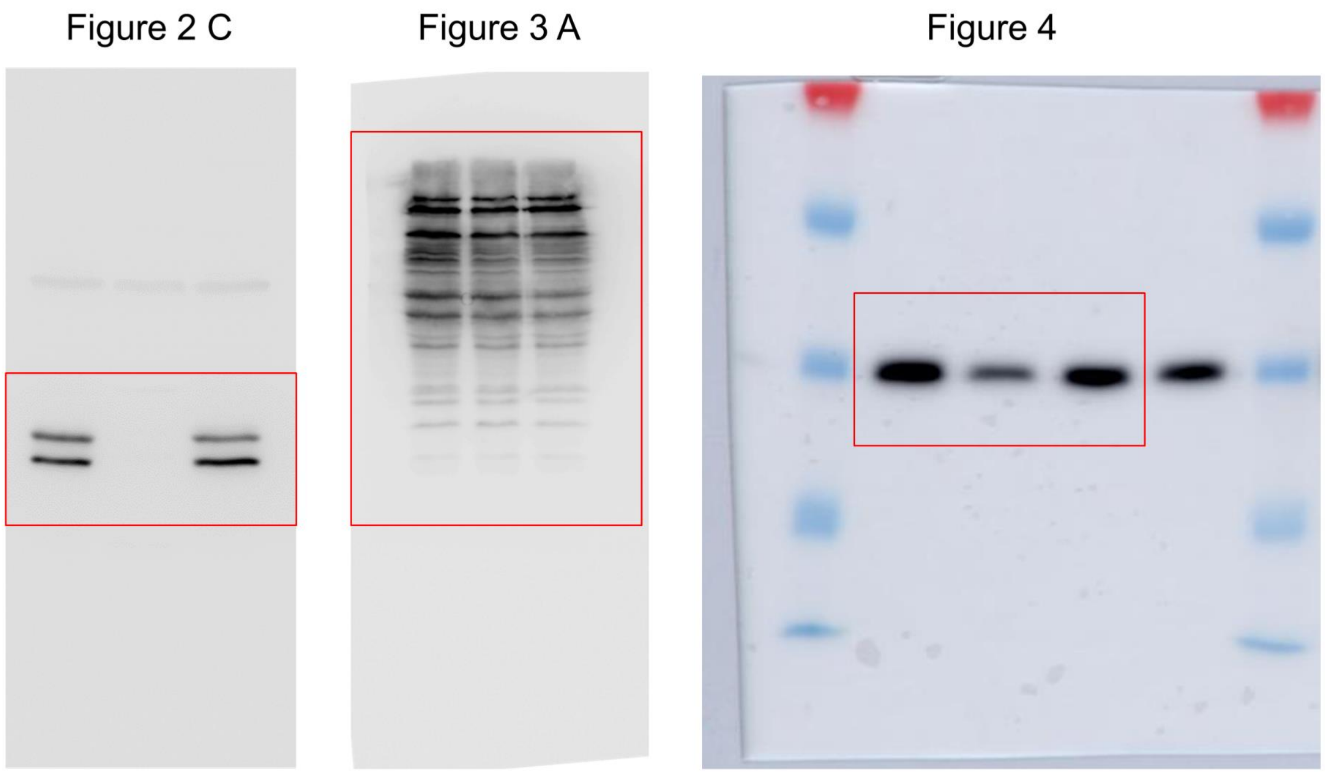

Figure S6. Full-length blots of immunoblots shown in Figure 2 C, Figure 3 A and Figure 4 in the main paper. The red frame marks the cropped area, respectively.

Figure 5

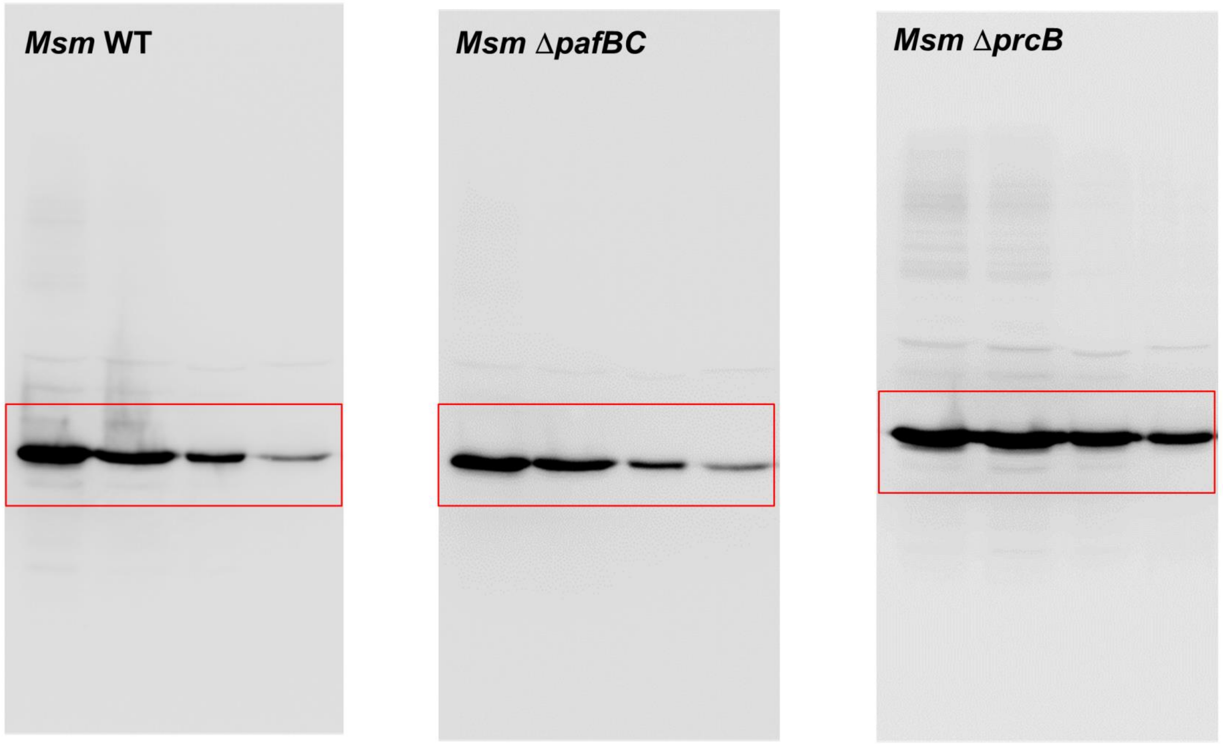

Figure S7. Full-length blots of immunoblots shown in Figure 5 in the main paper. The red frame marks the cropped area, respectively.

Figure 7 A

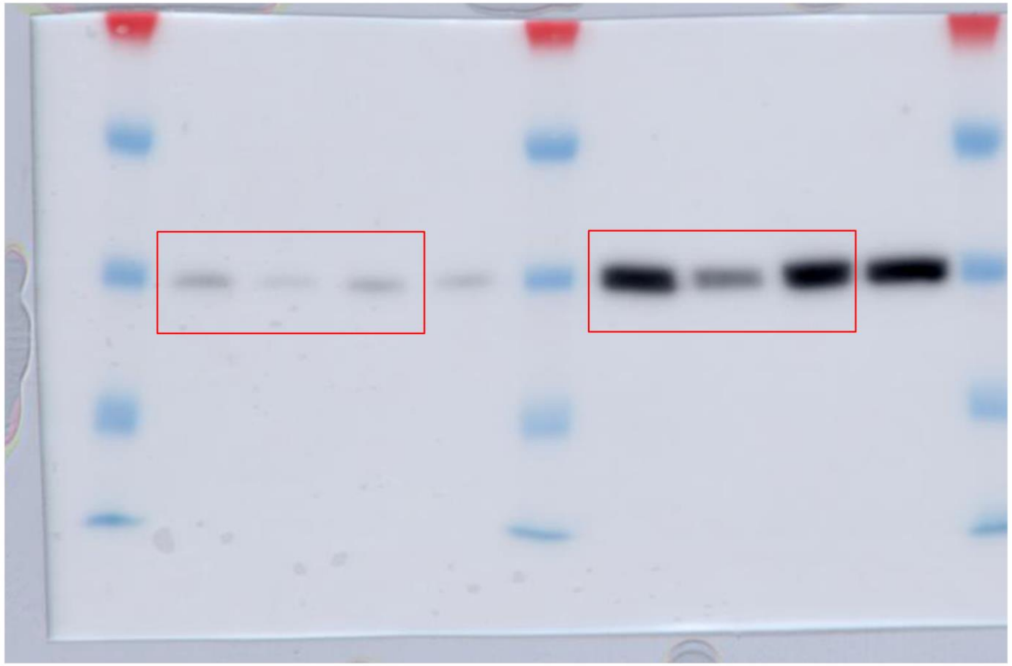

Figure 7 B

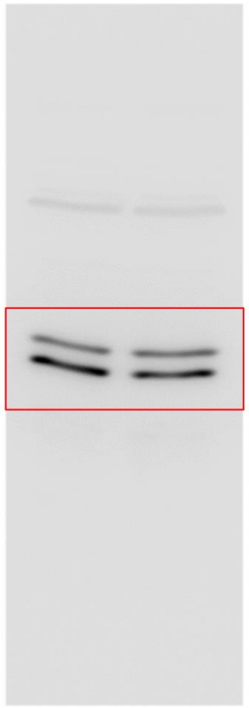

Figure S8. Full-length blots of immunoblots shown in Figure 7 A and Figure 7 B in the main paper. The red frame marks the cropped area, respectively.

P1 promoter consenus sequence - tTGTCRgtg (8 nt) TannnT-

Gene	Description[a]
**MSMEG_0005**	DNA gyrase subunit B
**MSMEG_0224**	O-methyltransferase
**MSMEG_1252 (1251)**	Uncharacterized protein
**MSMEG_1891**	Uncharacterized protein
**MSMEG_1941**[#]	DNA helicase
**MSMEG_1943 (1941)**	DNA helicase
**MSMEG_2679**[#]	Uncharacterized protein
**MSMEG_2723**	RecA
**MSMEG_3908 (3907)**	Uncharacterized protein
**MSMEG_4673**	ClpP1
**MSMEG_5002 (5004)**	Conserved hydrolase
**MSMEG_5511**[#]	von Willebrand factor, type A
**MSMEG_5680**[#]	Glyoxalase family protein
**MSMEG_5827**[#]	Glyoxalase family protein
**MSMEG_5935**[#]	RecQ

Table S1. PafBC might directly or indirectly regulate transcription of distinct genes *via* the LexA/RecA-independent promoter P1. The consensus sequence for the P1 promoter is displayed on top of the table[5-7]. The table shows proteins exhibiting affected cellular levels in the iTRAQ analysis of *Msm* Δ*pafBC*. The corresponding coding genes either display the P1 consensus motif in their upstream region or are organized in an operon with genes (shown in brackets) harbouring the sequence in the promoter region. **a**, descriptions according to the UniProt database; #, P1 motif in the upstream region was identified in this study.

Table S2. Oligonucleotides used in this study. Restriction sites are underlined. Overhangs for Gibson Assembly in lower case.

Name	Sequence 5' – 3'	Purpose
Bgl5pafBCMsm_fw	GACTAGATCTGGCCAGTCGGAACCCGTCC	Cloning pMCSΔ*pafBC*
EcoNde5pafBCMsrv	GCAATTCATATGGCAATTCCTTTTTTAGGGGCGATGACAAGGTTCATCAACCG	-"-
Eco3pafBCMsm_fw	GCAATTACCTAAAAAAGGCCGCCGAAGCCGCGTTGC	-"-
Nde3pafBCMsm_rv	CGTAATCATATGGGGGGTCTGCACAGGCTTGG	-"-
ProbeDpafBC_fw	GCGGCGCAGGCAAGGTGC	Amplification of probe for Southernblot analysis
ProbeDpafBC_rv	CGTCGAGCTGGCGGCCC	Amplification of probe for Southernblot analysis
recA fwd	GGCACTGAAGTTCTACGCCT	qRT-PCR analysis of *M. smegmatis recA*
recA rev	CATGTCGATGAGCGAACCCT	qRT-PCR analysis of *M. smegmatis recA*
pafA fwd	TCAGCAGGTCTTCGAGAACG	qRT-PCR analysis of *M. smegmatis pafA*
pafA rev	AGGTTGCCCGCTATCTGTTC	qRT-PCR analysis of *M. smegmatis pafA*
uvrB frd	GCCGTCGAGTACTTCGTCTC	qRT-PCR analysis of *M. smegmatis uvrB*
uvrB rev	CTCCATGTTGTAGCGGTCCT	qRT-PCR analysis of *M. smegmatis uvrB*
clgR fwd	GCATCGAGCGAGCTTCTCAG	qRT-PCR analysis of *M. smegmatis clgR*
clgR rev	CTCACGGGCGTCGTGTTC	qRT-PCR analysis of *M. smegmatis clgR*
16s fwd	GCGTTGTTCGTGAAAACTCA	qRT-PCR analysis of *M. smegmatis 16srRNA gene*
16s rev	CCTCCTGATATCTGCGCATT	qRT-PCR analysis of *M. smegmatis 16srRNA gene*
pafA_fw	CCAGGCTCAGCGCACCGTG	Analysis of *pafABC* co-transcrption

pafB_rv	GCCCGTCCCTCGTCGATCG	-“-
pafB_fw	CGATCGACGAGGGACGGGC	-“-
pafC_rv	GGGTTGGCCTGGAAGTACGGG	-“-
B_fwd	ATTTACCATGGCGAGAATCTTTATTTTCAGGGCCTCAGCGCGGTCTCCAAAGTCGAGCGGTTG	Cloning of *M. smegmatis pafB-Strep-pafC-Strep* in pMyC for protein purification
B_rev	AATAACCTAGGTCATCGTTCCCCTCCCGCG	-“-
C-fwd	ATTTACCATGGCGAGAATCTTTATTTTCAGGGCCTCAGCAGTCAGGTGTCGACGCGAC	-“-
C_rev	AATAACCTAGGTCATACGCAACGGGCATAGGC	-“-
Prom_recA_fw	GCTAAAGCTTGCTGTGCACGCGCCGTCC	Amplification 300 bp of *M.smegmatis recA* promoter region (EMSA)
Prom_recA_rv	TATCAAGCTTGATATCTTCCCATGGTGGTGCCTCTCCGAGTAGTC	Amplification 300 bp of *M.smegmatis recA* promoter region (EMSA)
Prom_pafA_fw	GCTAAAGCTTCGACGTCGGTCAGCAGGGGG	Amplification 300 bp of *M.smegmatis pafABC* promoter region (EMSA)
Prom_pafA_rv	GCTAAAGCTTATGCATCCATGGCATCGAGCTTACGGGTTGCTGCAC	Amplification 300 bp of *M.smegmatis pafABC* promoter region (EMSA)
Prom_sucB_fw	ATGCAAGCTTCCACCCATCTCTGCATGTACTGG	Amplification 300 bp of *M.smegmatis sucB* promoter region (EMSA)
Prom_sucB_rv	TATCAAGCTTATCGATTCCCATGGTGTGTTGACTCCCTCGGACGG	Amplification 300 bp of *M.smegmatis sucB* promoter region (EMSA)
Prom_groEL_fw	GCTAAAGCTTCGCCGCGGAACATCTGACG	Amplification 300 bp of *M.smegmatis groEL* promoter region (EMSA)
Prom_groEL_rv	GCTAAAGCTTATCGATGCCATGGCGAAGTGTTCCTCCGGATTGGG	Amplification 300 bp of *M.smegmatis groEL* promoter region (EMSA)
PrecAP1_rv	ATGTAAGCTTATGCATGATATCGGCCTTTTCGCGATCTGGGG	Cloning of promoter recA promoter GFP reporter
GFP_P1_fw	ATCTGATATCTCAAAGGGCGAGGAGCTGTTCAC	-“-
GFP_rv	GCTAATGCATTCACTTGTACAGCTCGTCCATGCCGT	Cloning of promoter *recA* P1-GFP and *recA* P2-GFP constructs in pMV361-*hyg-amp*

PrecAP2_fw	ATCAAAGCTTGGCCGCAGTGCCCTCGG	Cloning of promoter *recA* P2-GFP construct in pMV361-*hyg-amp*
PrecAP2_rw	AGA TAA GCT TAT GCA TCC ATG GGT CCG GTC GAA CTC TTC ACC AC	-"-
GFP_P2_fw	CAATCTTCATGACTACTCGGAGAGGCACCACCATGGGGTCGAAGGGCGAGGAG	-"-
pMyNTint-lin_fw	AAGCTTATCGATGTCGACGTAGTTAAC	-"-
pMyNTint-lin_rv	TGAGACCGCGTCACTTCTTTATC	-"-
pRecA_fw	gataaagaagtgacgcggtctcaGGCCGCAGTGCCCTCG	-"-
GFP_rv	cgtcgacatcgataagcttTCACTTGTACAGCTCGTCCATG	-"-
P2h2a_rv	tggaacacatcacttagctccatactgTGGACACGCCGAGCTG	-"-
P2h2a_fw	gagctaagtgatgtgttccaGTGGTGAAGAGTTCGACCG	-"-
P1h2a_rv	agctccatactgctgtagacctcGTCCGGTCGAACTCTTCAC	-"-
P1h2a_fw	gtctacagcagtatggagctCACGGCCAACCGATCG	-"-
His6recA_fw	ATCGCCATGGGAGCACAGCAGGCCCCAGATC	Cloning of *M. smegmatis* recA-His_6 in pMyC
His6recA_rv	TAATGATATCTCAGTGGTGGTGGTGGTGGTGGAAGTCAACCGGGGCTGGG	-"-
NcoPafBMsm_fw	GACTCCATGGGGGCGGTCTCCAAAGTCGAGCGG	Cloning of complementation plasmid pMV361-*pafBC*
HindPafCMsm_rv	GCTAAAGCTTTCATACGCAACGGGCATAGGCC	Cloning of complementation plasmid pMV361-*pafBC*

References

1 Bordoli, L. *et al.* Protein structure homology modeling using SWISS-MODEL workspace. *Nat Protoc* **4**, 1-13 (2009).

2 Weekes, D. *et al.* Crystal structure of a transcription regulator (TM1602) from *Thermotoga maritima* at 2.3 Å resolution. *Proteins* **67**, 247-252 (2007).

3 Wilson, K.P., Shewchuk, L.M., Brennan, R.G., Otsuka, A.J., Matthews, B.W. *Escherichia coli* biotin holoenzyme synthetase/bio repressor crystal structure delineates the biotin- and DNA-binding domains. *Proc.Natl.Acad.Sci.* **89**, 9257-9261 (1992).

4 Cherney, L.T., Cherney, M.M., Garen, C.R., Lu, G.J., James, M.N. Crystal structure of the arginine repressor protein in complex with the DNA operator from *Mycobacterium tuberculosis*. *J Mol Biol* **384**, 1330-1340 (2008).

5 Sievers F, Wilm A, Dineen DG, Gibson TJ, Karplus K, Li W, Lopez R, McWilliam H, Remmert M, Söding J, Thompson JD, Higgins D Fast, scalable generation of high-quality protein multiple sequence alignments using Clustal Omega. *Mol Syst Biol* **7,** Article number: 539, doi:10.1038/msb.2011.75 (2011).

6 Aravind, L., Anantharaman, V., Balaji, S., Babu, M. M. & Iyer, L. M. The many faces of the helix-turn-helix domain: transcription regulation and beyond. *FEMS Microbiol Rev* **29**, 231-262 (2005).

7 Edelhoch H. Spectroscopic determination of tryptophan and tyrosine in proteins. *Biochemistry* **6**(7), 1948-1954 (1967).

8 Pace CN, Vajdos F, Fee L, Grimsley G, Gray T. How to measure and predict the molar absorption coefficient of a protein. *Protein Sci* **4**(11), 2411-2423 (1995).

9 Movahedzadeh, F., Colston, M. J. & Davis, E. O. Determination of DNA sequences required for regulated *Mycobacterium tuberculosis* RecA expression in response to DNA-damaging agents suggests that two modes of regulation exist. *J Bacteriol* **179**, 3509-3518 (1997).

10 Gamulin, V., Cetkovic, H. & Ahel, I. Identification of a promoter motif regulating the major DNA damage response mechanism of *Mycobacterium tuberculosis*. *FEMS Microbiol Lett* **238**, 57-63 (2004).

11 Wang, Y., Huang, Y., Xue, C., He, Y. & He, Z. G. ClpR protein-like regulator specifically recognizes RecA protein-independent promoter motif and broadly regulates expression of DNA damage-inducible genes in mycobacteria. *J Biol Chem* **286**, 31159-31167 (2011).
